# Cuproptosis-related lncRNA JPX regulates malignant cell behavior and epithelial-immune interaction in head and neck squamous cell carcinoma via miR-193b-3p/PLAU axis

**DOI:** 10.1038/s41368-024-00314-y

**Published:** 2024-11-08

**Authors:** Mouyuan Sun, Ning Zhan, Zhan Yang, Xiaoting Zhang, Jingyu Zhang, Lianjie Peng, Yaxian Luo, Lining Lin, Yiting Lou, Dongqi You, Tao Qiu, Zhichao Liu, Qianting Wang, Yu Liu, Ping Sun, Mengfei Yu, Huiming Wang

**Affiliations:** Stomatology Hospital, School of Stomatology, Zhejiang University School of Medicine, Zhejiang Provincial Clinical Research Center for Oral Diseases, Key Laboratory of Oral Biomedical Research of Zhejiang Province, Cancer Center of Zhejiang University, Engineering Research Center of Oral Biomaterials and Devices of Zhejiang Province, Hangzhou, China

**Keywords:** Cancer, Biomarkers

## Abstract

The development, progression, and curative efficacy of head and neck squamous cell carcinoma (HNSCC) are influenced by complex interactions between epithelial and immune cells. Nevertheless, the specific changes in the nature of these interactions and their underlying molecular mechanisms in HNSCC are not yet fully understood. Cuproptosis, a form of programmed cell death that is dependent on copper, has been implicated in cancer pathogenesis. However, the understanding of cuproptosis in the context of HNSCC remains limited. In this study, we have discovered that cuproptosis-related long non-coding RNAs (CRLs) known as JPX play a role in promoting the expression of the oncogene urokinase-type plasminogen activator (PLAU) by competitively binding to miR-193b-3p in HNSCC. The increased activity of the JPX/miR-193b-3p/PLAU axis in malignant epithelial cells leads to enhanced cell proliferation, migration, and invasion in HNSCC. Moreover, the overexpression of PLAU in tumor epithelial cells facilitates its interaction with the receptor PLAUR, predominantly expressed on macrophages, thereby influencing the abnormal epithelial–immune interactome in HNSCC. Notably, the JPX inhibitor Axitinib and the PLAU inhibitor Palbociclib may not only exert their effects on the JPX/miR-193b-3p/PLAU axis that impacts the malignant tumor behaviors and the epithelial–immune cell interactions but also exhibit synergistic effects in terms of suppressing tumor cell growth and arresting cell cycle by targeting epidermal growth factor receptor (EGFR) and cyclin-dependent kinase (CDK4/6) for the treatment of HNSCC.

## Introduction

Head and neck squamous cell carcinoma (HNSCC) is a heterogeneous originating in the squamous epithelium of the mouth, oropharynx, larynx, and hypopharynx, accounting for approximately 90% of head and neck cancers, ranked the eighth most common malignancy worldwide.^1–3^ Surgery treatment, radiotherapy, and chemotherapy are the traditional effective therapeutic strategies for HNSCC patients; however, the benefit of these therapies is limited by intra-tumor heterogeneity.^4,5^ Even with the advance of immunotherapy in the past decade, the clinical utility of HNSCC has significantly improved, and the effect of immunotherapies is also affected by numerous drug resistance mechanisms.^6^ In order to reach an ideal efficacy endpoint, a more holistic interrogation of the complex interaction among cell populations in the tumor microenvironment (TME) and the underlying molecular mechanisms should be considered.^7^

Change in the mode of these interactions and their molecular mechanism in HNSCC might be related to cuproptosis. Cuproptosis is a recently discovered copper-dependent and -regulated new type of death closely related to mitochondrial respiration.^8^ Intracellularly, copper ions directly bind to the lipoylated components of the tricarboxylic acid cycle, which leads to the aggregation of lipoylated proteins and the downregulated expression of iron-sulfur cluster proteins, thereby inducing proteotoxic stress and eventually causing cell death.^9^ Numerous studies have shown that the disorder of copper metabolism is closely associated with many diseases, especially in the field of cancer.^10^ Several studies have confirmed that copper metabolism is related to tumorigenesis, and cancer cells have a higher demand for copper than normal cells.^11,12^ A variety of cancer types, such as breast cancer^13^ and cervical cancer,^14^ show increased copper content in tumor tissues and/or changes in copper distribution throughout the body. A similar phenomenon can occur in head and neck squamous cell carcinoma (HNSCC).^15^ Cuproptosis may function by suppressing cancer cell proliferation and inhibiting metastatic events, but the direct facilitating of the cuproptosis process can cause damage to adjacent normal cells.^16^ Therefore, there is an urgent need to find how cuproptosis influences the progression of HNSCC via its underlying mechanism and downstream target.

Long non-coding RNA (LncRNA) is a subgroup of ncRNA with transcripts longer than 200 nucleotides, which plays a crucial role in regulating chromatin dynamics, gene expression, growth, differentiation, and development.^17^ A large amount of LncRNAs have been identified to be closely related to tumor occurrence and metastasis through Genome-wide association studies (GWAS) of tumor samples, so that they may be adopted as early prognostic indicators of cancers.^18,19^ Previous studies have reported that cuproptosis-related lncRNAs (CRLs) predict the prognosis of cancer, including lung adenocarcinoma, colorectal cancer, and gastric cancer.^20–22^ CRLs may also be involved in the metabolic and immune pathways of HNSCC and can act as a potential therapeutic target of HNSCC.^23,24^ However, these studies obscure the cellular heterogeneity and cannot illustrate the specific mechanism of CRLs in different cells participating in HNSCC pathogenesis.

Here, we integrated single‐cell RNA-sequencing (scRNA-seq) analysis of 6 HNSCC tissues and bulk genomic information of 502 HNSCC tissues to comprehensively assess the underlying cellular distribution of CRL and its correlation with major non-immune and immune features in HNSCC. Survival, differentiation, and correlation analyses were exploited to determine the role of CRL. Using a random forest (RF) model, we selected the key lncRNA JPX among these CRLs and implemented a competing endogenous RNA (ceRNA) network. Subsequently, HNSCC characteristic malignant cells were identified and went through enrichment analysis, and further experimental verification was carried out to further assess the potential molecular mechanisms. Moreover, we revealed an interaction mode of aberrant epithelial cells and immune cells in HNSCC and identified populations of epithelial cells influencing macrophage behavior in HNSCC. Considering the cross-resistance toward the traditional anti-HNSCC drugs, we aimed to present a curative combined therapeutic strategy not routinely used as HNSCC therapeutic compounds in clinics in this study. Thereby, we propose the combined of JPX and its downstream gene urokinase-type plasminogen activator (PLAU) inhibitors as a novel therapeutic strategy to target HNSCC and provide new insights into precision oncology.

## Results

### Cell type diversity and cuproptosis specificity in HNSCC

**Fig. 1:**
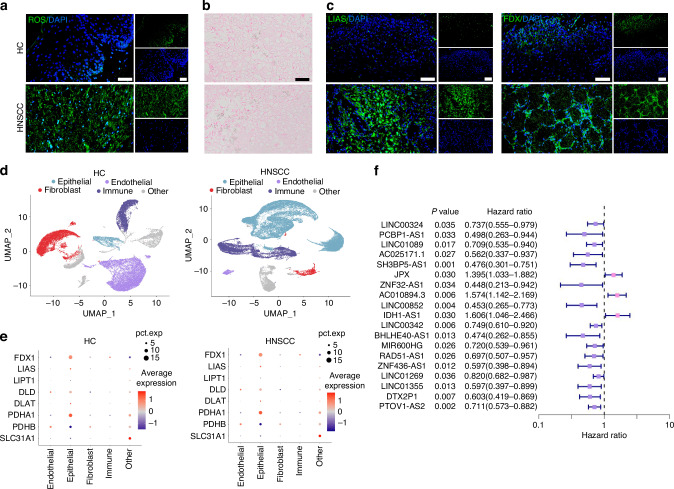
Cuproptosis-related lncRNAs (CRLs) and major cell types associated with HNSCC. **a** DCFH-DA fluorescence image for HNSCC clinical samples. *n* = 7 per group. Quantitative analysis of intensity is also shown. 40×, scale bar = 50 μm. **b** Rubeanic acid copper staining for HNSCC clinical samples. 40×, scale bar = 50 μm. **c** Immunofluorescence images for the localization and the expression of LIAS and FDX1 in HNSCC clinical samples. *n* = 7 per group. Quantitative analysis of intensity is also shown. 40×, scale bar = 50 μm. **d** UMAP representation of major cell types identified by scRNA-seq in HC (left) and HNSCC (right). **e** Expression distribution of cuproptosis-related genes (CRGs) in major cell populations. **f** Univariate Cox regression analysis of 19 HNSCC-related prognostic CRLs

Cuproptosis is characterized by lipid peroxidation production, excessive accumulation of copper ions, and abnormal expression of cuproptosis-related genes. We measured the intracellular reactive oxygen species (ROS) by DCFH-DA fluorescent probe in HNSCC samples and found that the ROS content was much higher in HNSCC tissue samples than in HC tissue samples (Fig. 1a). Additionally, rubeanic acid copper staining indicated that HNSCC tissues remained similar copper ion content with HC tissue (Fig. 1b). On the contrary, immunofluorescence results showed that the expression levels of the anti-cuproptosis protein lipoic acid synthetase (LIAS) and ferredoxin 1 (FDX1) were upregulated in HNSCC tissue sample (Fig. 1c and Supplementary Fig. S1a). These findings supported the hypothesis that the abnormal expression of cuproptosis-related genes was the most important factor affecting cuproptosis in HNSCC.

To further probe for the cuproptosis-related gene expression specificity in different cell types, we performed scRNA sequencing data analysis. After quality control to remove low-quality cells expressing high mitochondrial gene signatures and exclude doublets, our dataset included 93 345 core cells for subsequent analysis (Supplementary Fig. S1b–j). The core cells were classified into 4 major cell compartments by the UMAP algorithm comprised of epithelial, endothelial, fibroblast, and immune cells (Fig. 1d). Analysis of the transcriptomic signatures for the major cell types documented differential expression of subcluster-defining genes for the 4 major cell compartments (Supplementary Fig. S1h). Representation of these 4 main cell types was different between HNSCC and healthy control (HC), as the HNSCC contained expanded immune and epithelial compartments (Supplementary Fig. S1g). Therefore, we surmised that these two compartments of cells play a crucial role in the occurrence and development of HNSCC. To explore the cuproptosis specificity among different cell populations in HNSCC, we selected ten hub cuproptosis-related genes (CRGs) by the PPI database and visualized the expression levels of these hub CRGs in the identified cell populations of HNSCC. The results indicated that CRGs were significantly modulated in the epithelial cell compartment of HNSCC, but the concrete mechanism remains unclear (Fig. 1e).

### TME characteristics of two HNSCC subtypes based on prognostic CRLs

A total of 118 CRLs were identified by co-expression analysis with CRGs (*R* > 0.4, FDR < 0.001) (Supplementary Fig. S2a). Then, 19 HNSCC-related prognostic CRLs were selected by univariate Cox regression analysis for the following study (Fig. 1f). Within the 19 prognostic CRLs, 11 CRLs were found to be significantly up- or downregulated in HNSCC cohort (logFC > 0, FDR < 0.05) (Supplementary Fig. S2b).

**Fig. 2:**
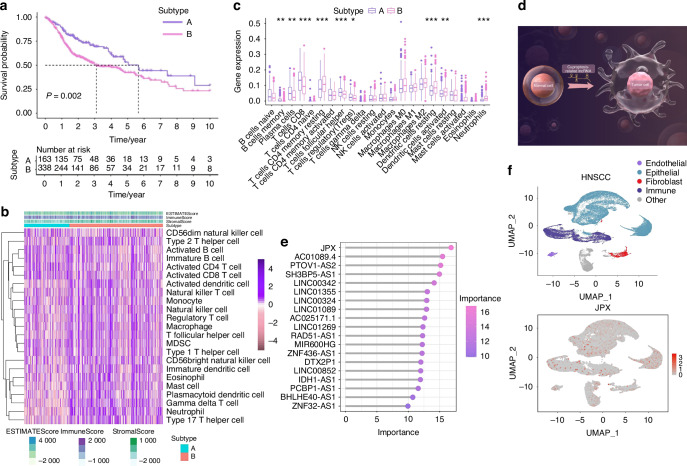
JPX is notably more concentrated within epithelial compartments. **a** Kaplan–Meier curves for differential survival of 2 CRL clusters. **b** Heat map of immune cells in 2 CRL clusters. **c** Immune cell-related KEGG enrichment levels comparison between 2 CRL clusters. **d** The illustration showed that CRLs acted as pivotal nodes in the molecular mechanism of HNSCC, by Figdraw. HC, healthy control; HNSCC, head, and neck squamous cell carcinoma. **e** 19 CRLs were identified as finial markers with importance > 10. **f** Expression of JPX is shown in UMAP. Wilcoxon signed-rank test: *P < 0.05, **P < 0.01, and ***P < 0.001

We conducted an unsupervised consensus analysis to understand the molecular heterogeneity of HNSCC from the perspective of CRLs. The results indicated that *k* = 2 was more reasonable, and all the samples were divided into two HNSCC subtypes (Subtype A and Subtype B), with less correlation between the two subtypes (Supplementary Fig. S3a). We compared the overall survival among the two subtypes of HNSCC patients via the K-M survival analysis to detect whether there was an association between the different subtypes and clinical outcomes. Overall survival of Subtype A was significantly longer than that of Subtype B (*P* = 0.002) (Fig. 2a).

Then, we further investigated whether CRL-based HNSCC subtypes showed different immune patterns, and the results indicated that Subtype A showed a distinctly different immune cell landscape and chemokine categories than Subtype B (Fig. 2b, c and Supplementary Fig. S3d, e, g, h). Among them, suppressive immune cells such as regulatory T cells and myeloid-derived suppressor cells (MDSC) were significantly reduced in Subtype B. Next, we compared the expression of immune checkpoint molecules between two HNSCC subtypes and found that out of ten differentially expressed immune checkpoints, CD276, CD47, PDCD1LG2, PVR had a higher expression in Subtype B (Supplementary Fig. S3f). Furthermore, tumor angiogenesis and extracellular matrix-related pathways and -related genes were both more enriched in Subtype B (Supplementary Fig. S3b, c). These results revealed that the key CRLs could regulate the TME characteristics of HNSCC. The results indicated that CRLs acted as pivotal nodes in the molecular mechanism of HNSCC (Fig. 2d), but the concrete mechanism remains to be explored.

### JPX regulates the malignant cell behaviors of CAL27 cells

**Fig. 3:**
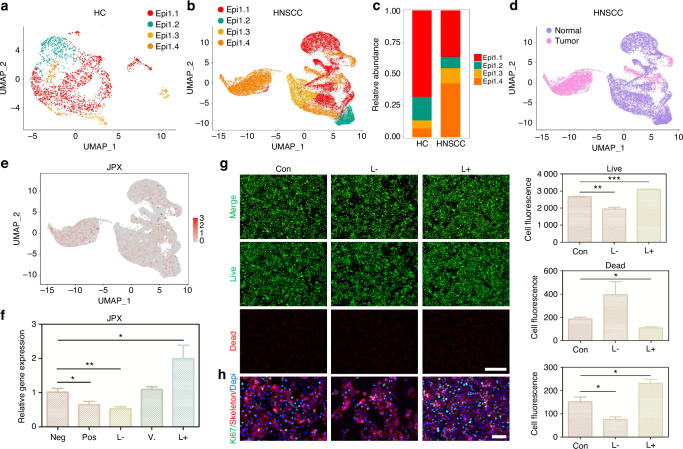
JPX regulates the malignant cell behaviors of CAL27 cells. **a** UMAP of epithelial cell populations in HC. **b** UMAP of epithelial cell populations in HNSCC. **c** Proportion plots of epithelial cell populations in HC and HNSCC. **d** UMAP plots comparing normal and malignant epithelial cells in HNSCC. **e** Expression of JPX is shown in UMAP of epithelial cell populations in HNSCC. **f** The relative expression levels of JPX in CAL27 cells with JPX knockdown or overexpression. **g** Live/dead staining of CAL27 cells with JPX knockdown or overexpression. Quantitative analysis of intensity is also shown. 20×, scale bar = 150 μm. **h** Ki-67 cells staining of CAL27 cells with JPX knockdown or overexpression. Quantitative analysis of intensity is also shown. 40×, scale bar = 50 μm. Con, control group; neg, empty vector group; pos, positive control group; L-, JPX knockdown group; V., vehicle group; L+, JPX overexpressed group. Wilcoxon signed-rank test: *P < 0.05, **P < 0.01, and ***P < 0.001

**Fig. 4:**
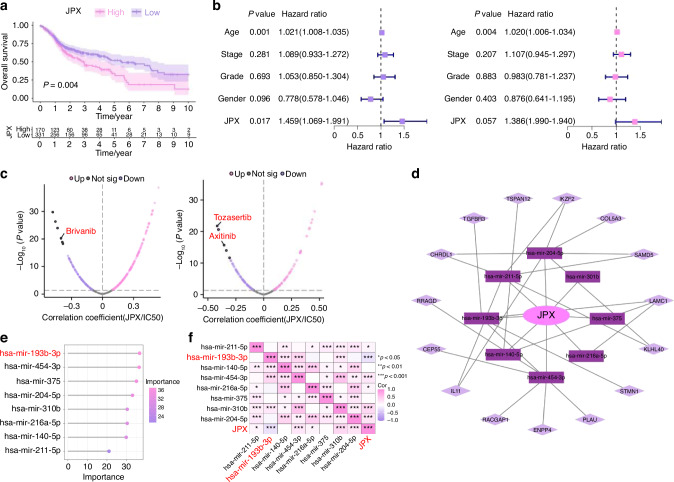
Functional analysis of JPX. **a** Kaplan–Meier Curves for differential survival of JPX high-expression and JPX low-expression subgroups. **b** Cox regression analysis of age, stage, grade, gender, and JPX. **c** Drug sensitivity analysis of JPX high-expression and JPX low-expression subgroups. **d** JPX-based ceRNA network. **e** 8 miRNAs were identified as finial markers with importance > 24. **f** Spearman’s correlation coefficient analysis of the miRNAs and JPX. Spearson correlation analysis: *P < 0.05, **P < 0.01, and ***P < 0.001

The 11 CRLs that expand in cancer were included for the Random Forest model construction (Supplementary Fig. S3i, 4b). We found that JPX was the most decisive in both the external test set and the training set (Fig. 2e and Supplementary Fig. S4a-e). Hence, we chose JPX for subsequent UMAP projection and found that JPX is more significantly concentrated in epithelial compartments of the HNSCC atlas compared to HC (Fig. 2f and Supplementary Fig. S4f). Focusing on one of the major cell types that expand in HNSCC, we reclustered the only cells identified as epithelial cells to search for subpopulations and obtained a more refined view of this population (Fig. 3a, b and Supplementary Fig. S5a, b). The results showed that Epi 1.4 was significantly expanded in HNSCC than HC and dominated the interaction with other epithelial cell subtypes (Fig. 3c and Supplementary Fig. S5d–i). Analysis of the transcriptomic signatures for the epithelial cell subtypes documented differential expression of their cell-defining genes. The heatmap showed the expression levels of the top 5 DEGs of each cell population. The top 5 DEGs of Epi 1.4 included efferocytosis-related gene (SLC2A1) and oxidation-reduction-related genes (AKR1C2 and AKR1C3) (Supplementary Fig. S5c). The cancer cell phenotype was determined by copy number variation (CNV) analysis and keratin expression, and Epi 1.4 contained substantial malignant epithelial cells (Fig. 3d). Compared to the normal epithelial cell clusters, the malignant epithelial cell cluster showed a higher correlation with focal adhesion, glycolysis, ECM organization, and NF-κB signaling (Supplementary Fig. S6a, b). Interestingly, the overlay of JPX distribution onto epithelial cell subpopulation projections indicated that JPX was more likely to distribute in HNSCC atlas rather than HC and mainly expressed in clusters of Epi 1.4 and malignant epithelial cells (Fig. 3e and Supplementary Fig. S5j). We pursued further cytology experiments to determine whether the CRL JPX would mediate the abnormal behaviors of malignant epithelial cells. Primarily, JPX was knockdown or overexpressed in CAL27 cells with siRNA segments targeting JPX or JPX overexpression plasmid (Fig. 3f). Low expression of JPX increased cell death in CAL27 cells, as detected by live-death staining (Fig. 3g). The results of *Ki-67* staining demonstrated that knockdown of JPX inhibited CAL27 cell proliferation. The *Ki-67* expression level in L- group was 1.8 times lower than that in control group (Fig. 3h). On the contrary, overexpression of JPX produced the opposite result, confirming that the malignant cell behaviors of CAL27 cells could be inhibited by reducing the expression level of JPX. We then divided HNSCC samples into JPX high-expression and JPX low-expression subtypes, and survival analysis showed that JPX expression was negatively correlated with overall survival (Fig. 4a). Subgroup analysis was stratified by age (>60, ≤60), TNM stage (I-II, III-IV), Grade (I-II, III-IV), gender (male, female) and JPX expression level. An internal verification was performed by comparing the riskScore of the different subgroups using the Wilcoxon signed-rank test. JPX expression level has the most important impact on survival in both subgroups (Fig. 4b). The related differentially expressed genes of JPX are shown in Supplementary Fig. S7a. The GO enrichment analysis, KEGG pathway analysis, and GSEA analysis results indicated that JPX was associated with keratinization, inflammation, proliferation, and invasion of HNSCC (Supplementary Fig. S7b, c). Then, we draw a mutational landscape to display the differentially mutated genes and their mutation types. Proved to be the high-frequency mutated gene in JPX high-expression subgroup, NOTCH1 and CASP8 might mediate the aggressive cell behaviors induced by JPX (Supplementary Fig. 7d). Subsequently, we performed a correlation analysis between JPX and predicted IC50 drug data from the GDSC, CTRP and PRISM databases and found that JPX high-expression HNSCC subclass was more sensitive to Tozasertib, Brivanib and Axitinib. (Fig. 4c and Supplementary Fig. S7e).

### The overexpression of miR-193b-3p relieved JPX-induced abnormal cell behaviors of CAL27 cells

**Fig. 5:**
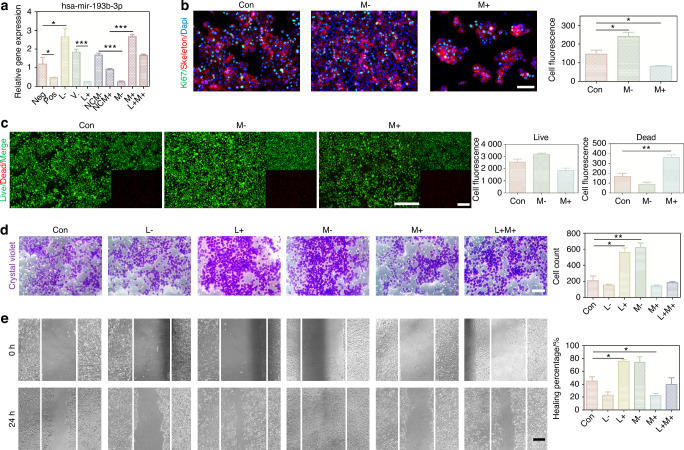
The overexpression of miR-193b-3p relieved JPX-induced abnormal cell behaviors of CAL27 cells. **a** The relative expression levels of miR-193b-3p in CAL27 cells with JPX/miR-193b-3p knockdown or overexpression. **b** Ki-67 cells staining of miR-193b-3p knockdown or overexpressed in CAL27 cells. Quantitative analysis of intensity is also shown. 40×, scale bar = 50 μm. **c** Live/dead staining of miR-193b-3p knockdown or overexpressed in CAL27 cells. Quantitative analysis of intensity is also shown. 20×, scale bar = 150 μm. **d** Migration assays in CAL27 cells with JPX/miR-193b-3p knockdown or overexpression. Quantitative analysis is also shown. 40×, scale bar = 50 μm. **e** Wound healing in CAL27 cells with JPX/miR-193b-3p knockdown or overexpression. Quantitative analysis of healing percentage is also shown. 10×, scale bar = 200 μm. Con control group, neg empty vector group, pos positive control group, L- JPX knockdown group, V. vehicle group, L+ JPX overexpressed group, NCM- miR-193b-3p inhibitor empty plasmid group, NCM+ miR-193b-3p mimic empty plasmid group, M- miR-193b-3p knockdown group, M+ miR-193b-3p overexpressed group, L+M+ JPX overexpressed and miR-193b-3p overexpressed group. Wilcoxon signed-rank test: *P < 0.05, **P < 0.01, and ***P < 0.001

**Fig. 6:**
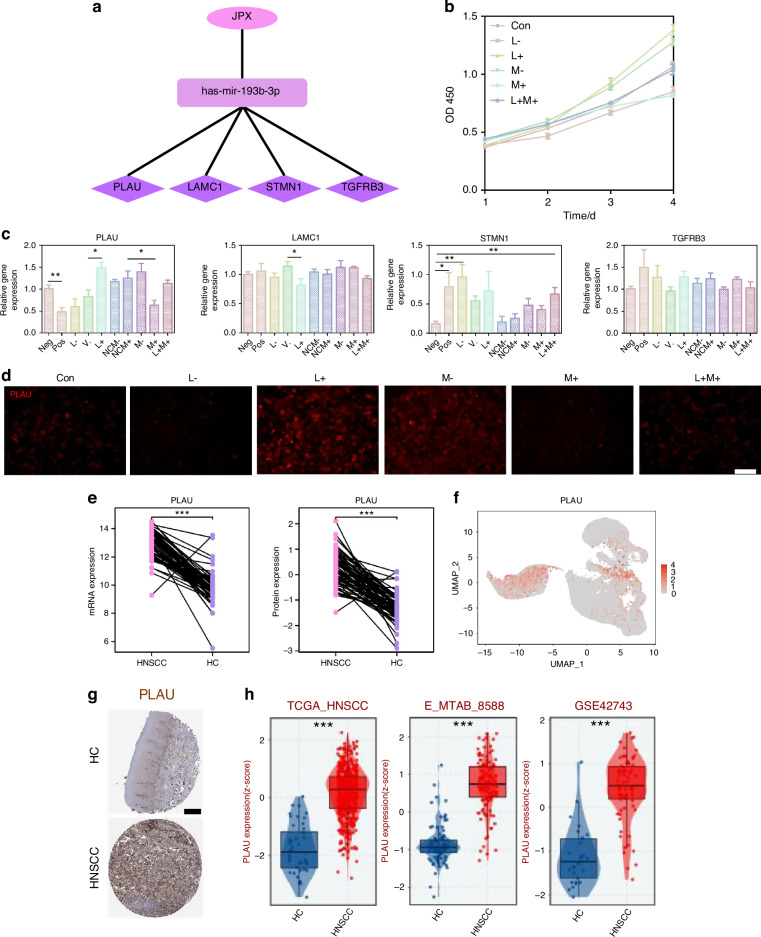
JPX upregulates the expression level of PLAU by inhibiting miR-193b-3p. **a** DEGs genes downstream of miR-193b-3p include PLAU, LAMC1, STMN1, and TGFBR3. **b** CCK-8 assay of CAL27 cells with JPX/miR-193b-3p knockdown or overexpression. **c** The relative expression levels of PLAU, LAMC1, STMN1, and TGFBR3 in CAL27 cells with JPX/miR-193b-3p knockdown or overexpression. **d** Immunofluorescence images for the localization and the expression of PLAU in CAL27 cells with JPX/miR-193b-3p knockdown or overexpression. 40×, scale bar = 50 μm. **e** Protein expression and mRNA expression of RRM2 based on CPTAC database. **f** Expression of PLAU is shown in UMAP of epithelial cell populations in HNSCC. **g** Available pathology images of PLAU localization and expression in HC and HNSCC were obtained from the HPA database. **h** Different expression levels of PLAU between the HC and HNSCC groups. Con control group, neg empty vector group, pos positive control group, L- JPX knockdown group, V. vehicle group, L+ JPX overexpressed group, NCM- miR-193b-3p inhibitor empty plasmid group, NCM+ miR-193b-3p mimic empty plasmid group, M- miR-193b-3p knockdown group, M+ miR-193b-3p overexpressed group, L+M+ JPX overexpressed and miR-193b-3p overexpressed group. Wilcoxon signed-rank test: *P < 0.05, **P < 0.01, and ***P < 0.001

We constructed a ceRNA network to further investigate how the lncRNA JPX regulated pivotal mRNA expressions by targeting miRNAs, ultimately leading to the aggressive cell behaviors in HNSCC (Fig. 4d). A total of 8 miRNAs and 15 mRNAs were included in the JPX-based ceRNA network. We used the 8 miRNAs for the Random Forest model construction and found that miR-193b-3p was the most effective miRNA in both the external test set and the training set (Fig. 4e and Supplementary Fig. S8a). The Spearman’s correlation coefficient analysis of miR-193b-3p showed that miR-193b-3p functioned relatively independently from the other miRNAs and was negatively correlated with JPX (Fig. 4f). Therefore, we further focused on the downstream mechanism of miR-193b-3p in HNSCC. Downregulation of miR-193b-3p in JPX-overexpressed CAL27 cells and upregulation of miR-193b-3p in JPX-knockdown CAL27 cells were verified by RT-qPCR, indicating that JPX was located upstream of miR-193b-3p in the regulatory network (Fig. 5a). Results of *Ki-67* staining, CCK8 assay and live-death staining indicated that hyperexpressive miR-193b-3p relieved JPX overexpression-induced abnormal cell proliferation of CAL27 cells (Figs. 5b, c and 6b). We also found that hyperexpressive miR-193b-3p effectively inhibits the migration of CAL27 cells in transwell assay (Fig. 5d). Consistent with this, in wound healing assay, miR-193b-3p overexpressing caused the healing percentage decline from 45% to 22% (Fig. 5e). CAL27 cells modified with both JPX overexpression and miR-193b-3p overexpression exhibited the comparable cell behaviors as the control group (Fig. 5a–e).

### JPX upregulates the expression level of PLAU by inhibiting miR-193b-3p

**Fig. 7:**
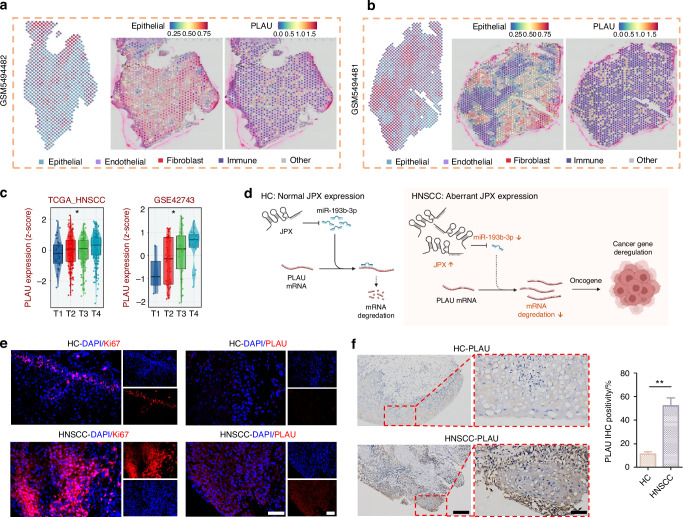
JPX/miR-193b-3p/PLAU signaling axis entwined with malignant cell behaviors and drug resistance in HNSCC. **a**, **b** The spatial distribution of epithelial cells and PLAU. **c** The expression levels of PLAU with T-stage. Kruskal−Wallis test: *P < 0.05, **P < 0.01, and ***P < 0.001. **d** Schematic diagram of the mechanism by which the JPX/miR-193b-3p/PLAU axis mediates HNSCC. **e** Immunofluorescence images for the localization and the expression of Ki-67 and PLAU in HNSCC clinical samples. *n* = 7 per group. 40×, scale bar = 50 μm. **f** Immunohistochemical images for the localization and the expression of PLAU in HNSCC clinical samples. *n* = 7 per group. Quantitative analysis of positive areas is also shown. 10×, scale bar = 200 μm. 40×, scale bar = 50 μm. Wilcoxon signed-rank test: *P < 0.05, **P < 0.01, and ***P < 0.001

**Fig. 8:**
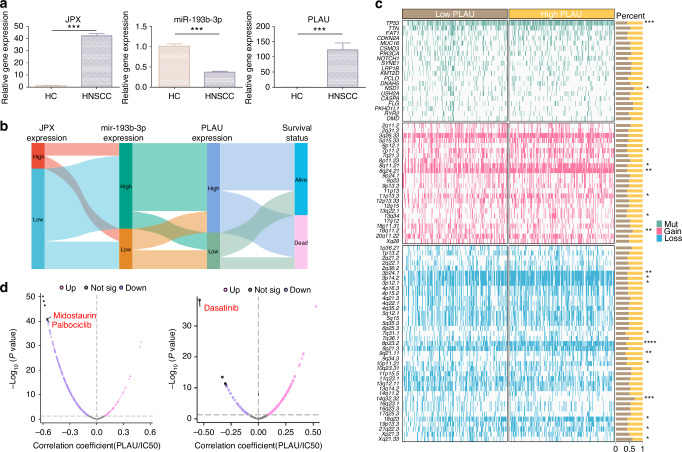
Drug sensitivity analysis of PLAU. **a** The relative expression levels of JPX, miR-193b-3p, and PLAU in HNSCC clinical samples. *n* = 7 per group. **b** Sankey diagram showing JPX, miR-193b-3p, PLAU expression, and clinicopathologic characteristics. **c** Somatic mutation characteristics of PLAU high-expression and PLAU low-expression subgroups in HNSCC. **d** Drug sensitivity analysis of PLAU high-expression and PLAU low-expression subgroups. Wilcoxon signed-rank test: *P < 0.05, **P < 0.01, and ***P < 0.001

In order to determine the downstream genes of miR-193b-3p, we detected the expression level of PLAU, LAMC1, STMN1 and TGFBR3, which were contained in the ceRNA network (Fig. 6a). Among them, only PLAU showed an enhanced expression trend after JPX overexpression or miR-193b-3p knockdown, and an inhibited expression trend after JPX knockdown or miR-193b-3p overexpression in line with our previous prediction (Fig. 6c). Consistently, immunofluorescence results also indicated that the expression level of PLAU is downregulated in CAL27 cells after JPX knockdown or miR-193b-3p overexpression (Fig. 6d and Supplementary Fig. S8b). In addition, the RT-qPCR results confirmed that the difference in expression of PLAU in HNSCC and HC was the most significant, compared to LAMC1, STMN1, and TGFBR3 (Fig. 8a and Supplementary Fig. S9b). This may imply that PLAU was the critical functional target of JPX in malignant epithelial cells. The mRNA expression and protein expression of PLAU were higher in HNSCC than in HC, based on the CPTAC database (Fig. 6e). Besides, PLAU expression was higher in HNSCC in the external test set and the training set from TCGA-HNSCC cohort (Fig. 6h and Supplementary Fig. S8c). Analysis of immunohistochemistry based on the online platform HPA (https://www.proteinatlas.org/) also suggested that PLAU was highly expressed in HNSCC malignant tissue samples (Fig. 6g). Based on PLAU expression in our scRNA-seq dataset, we found that PLAU was mainly expressed in the Epi 1.4 and malignant epithelial cell cluster (Fig. 6f and Supplementary Fig. S8d). Subsequently, we integrated the spatial transcriptome data with the scRNA-seq dataset. We mapped the five types of cells and PLAU to HNSCC tissue sections, which showed that the PLAU mapping range was essentially the same as that of the epithelial cell mapping range, suggesting that PLAU was predominantly expressed in the epithelial cell, in agreement with our previous prediction (Fig. 7a, b and Supplementary Fig. S8e, f). K-M survival analysis showed that patients with high PLAU expression had a poorer clinical prognosis (Supplementary Fig. S9a). Additionally, patients with higher T stages of HNSCC had higher PLAU expression (Fig. 7c). So, we came to the conclusion that abnormal activation of JPX/miR-193b-3p/PLAU signaling axis resulted in the aberrant cell proliferation, migration, and invasion of the malignant epithelial cells in HNSCC (Fig. 7d).

For further confirmation, we performed *Ki-67* staining in clinical tissue samples, and the results suggested that the cell proliferation in HNSCC tissue samples was 7.6 times more vigorous than that in HC tissue samples (Fig. 7e and Supplementary Fig. S9d). Meanwhile, the expression level of PLAU in tissue samples was examined by immunofluorescence and immunohistochemistry. In line with expectations, PLAU was highly expressed in all the HNSCC samples tested (Fig. 7e, f and Supplementary Fig. S9d). Additionally, the results of RT-qPCR showed that JPX and PLAU were 35.4 and 184.5 times, respectively, more highly expressed in HNSCC tissue samples than HC, while miR-193b-3p expressed 2.74 times lower (Fig. 8a). Expression of JPX, miR-193b-3p and PLAU, and clinical survival status were described using a Sankey-diagram. The results reconfirmed that JPX high-expression led to a higher proportion of miR-193b-3p low expression and PLAU high-expression, and the PLAU high-expression samples higher proportion of deaths (Fig. 8b). We draw a mutational landscape to display the differentially mutated genes and their mutation types. NSD1, a tumor-related gene encoding methyltransferase, was observed to be the high-frequency mutated gene in PLAU high-expression subgroup, while TP53, a common suppressor gene, was found to be the high-frequency mutated gene in PLAU low-expression subgroup (Fig. 8c). Subsequently, we performed a correlation analysis between PLAU and predicted IC50 drug data from the GDSC, CTRP and PRISM databases and found that PLAU expression significantly enhanced the chemosensitivity of Dasatinib, Midostaurin and Palbociclib in HNSCC (Fig. 8d and Supplementary Fig. S10a).

### PLAU mediates the epithelial–immune interactome in HNSCC

**Fig. 9:**
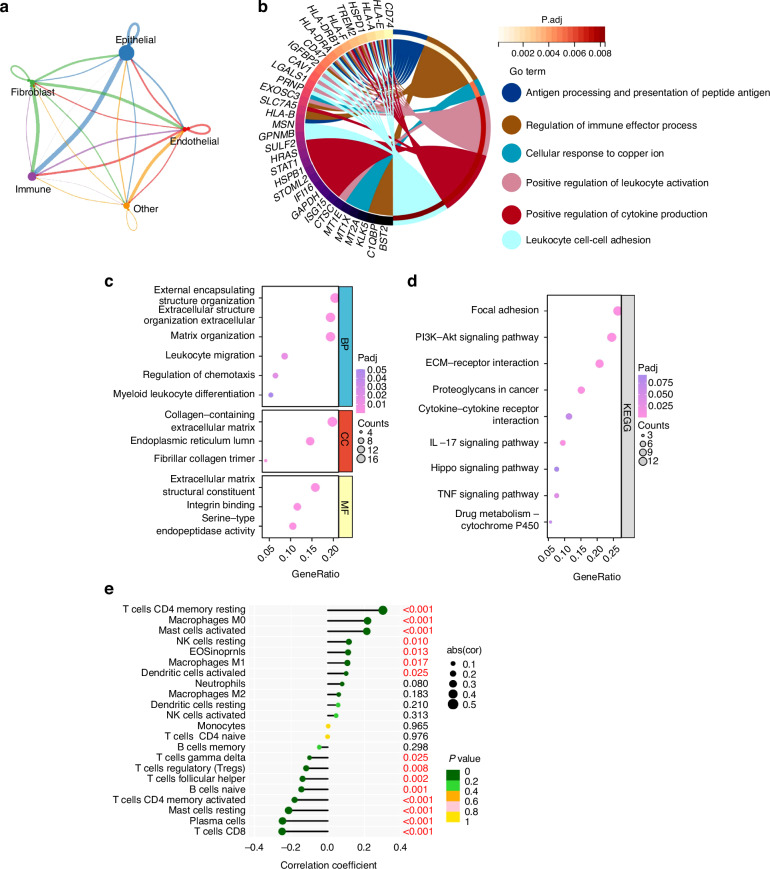
Cellchat analysis in HNSCC. **a** Strength of ligand-receptor interactions between cell population pairs of HNSCC based on CellChat analysis. Edge width is proportional to the number of ligand-receptor pairs. Circle sizes are proportional to the number of cells per cluster. **b** Chord diagrams of pathway enrichment. **c** Dot plots of GO enrichments associated with PLAU in HNSCC. **d** Dot plots of KEGG pathways associated with PLAU in HNSCC. **e** The abundance of immune cells varied a lot between PLAU high-expression and PLAU low- expression subgroups

Based on CellChat analysis within the 4 major cell compartments in our scRNA-seq dataset, we found that epithelial cells dominated the interaction with other cell compartments, and the largest number of interactions between epithelial cells and immune cells was noted in HNSCC (Fig. 9a and Supplementary Fig. S1j). From Fig. 9b, the enrichment of GO analysis of malignant epithelial cell clusters of epithelial cells listed antigen processing and presentation of peptide antigen, regulation of immune effector process, cellular response to copper ion, positive regulation of leukocyte activation, positive regulation of cytokine production and leukocyte cell–cell adhesion. This would indicate that epithelial cells shift to an inflammatory state in HNSCC, implying an interaction between malignant epithelial cells and immune cells in spite of the unclear main function bearer of this process. GO enrichment analysis, KEGG pathway analysis, and GSEA analysis results indicated that PLAU was associated with aggressive tumor behavior and tumor immune microenvironment (Fig. 9c, d and Supplementary Fig. S11). Key genes related to immune function, including MMP14, ITGA6, IL-1A, TGFB1, CSF2, etc., were involved in the PLAU downstream pathways interaction network (Supplementary Fig. S9c). PLAU was positively correlated with the abundance of several immune cells analyzed (Fig. 9e). Therefore, it is speculated that PLAU may mediate the functional connection between malignant epithelial cells and immune cells.

**Fig. 10:**
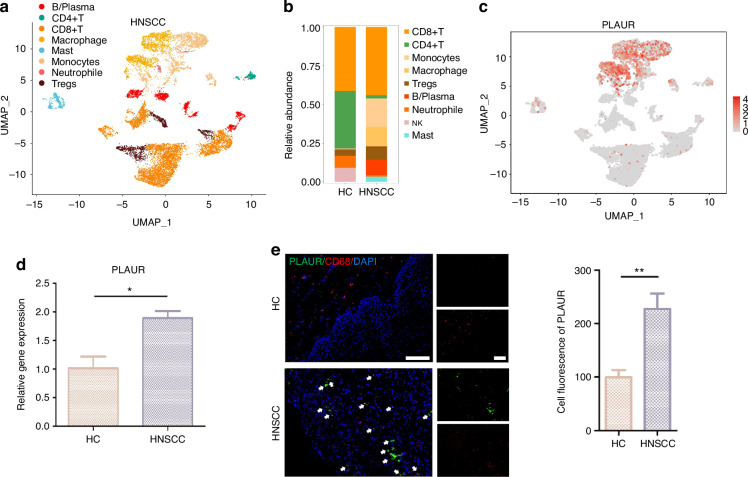
PLAU mediates the epithelial–immune interactome in HNSCC. **a** UMAP of immune cell populations in HNSCC. **b** Proportion plots of immune cell populations in HC and HNSCC. **c** Expression of PLAUR in UMAP of immune cell populations in HNSCC. **d** The relative gene expression levels of PLAUR in HNSCC clinical samples. *n* = 7 per group. **e** Immunofluorescence images of PLAUR (green) and CD68 (red, characterizing macrophages) in HNSCC clinical samples. *n* = 7 per group. Quantitative analysis of intensity is also shown. 20×, scale bar = 150 μm. Wilcoxon signed-rank test: *P < 0.05, **P < 0.01, and ***P < 0.001

**Fig. 11:**
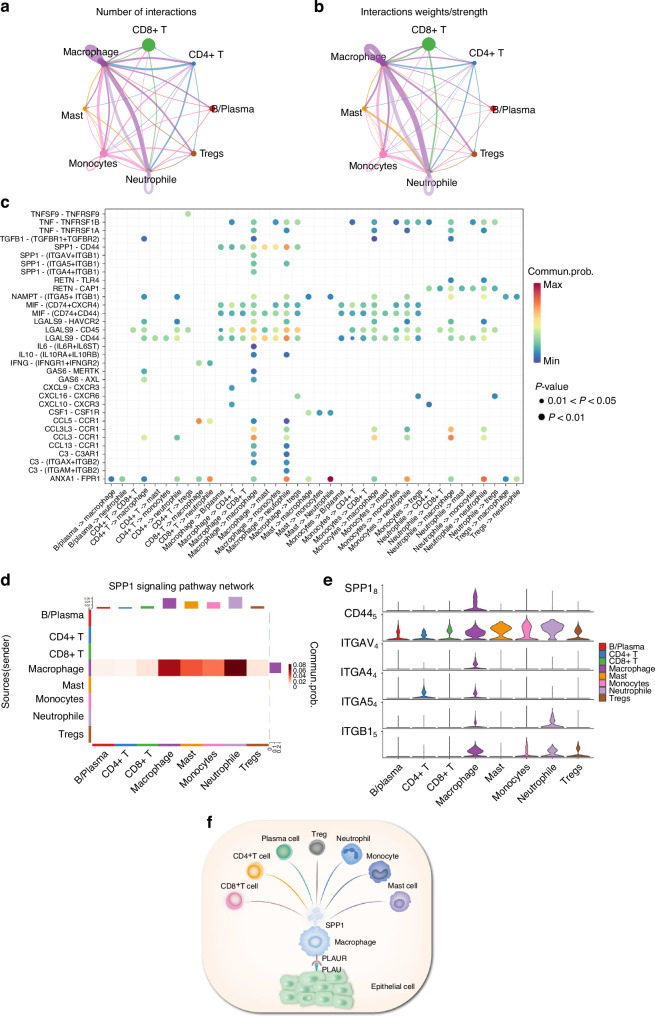
The intercell communication within the immune cells is mediated by SPP1 regulation. **a** Number of ligand-receptor interactions between immune cell population pairs of HNSCC based on CellChat analysis. **b** Strength of ligand-receptor interactions between immune cell population pairs of HNSCC based on CellChat analysis. **c** Bubble plots of the significant differentially expressed ligand-receptor pairs of HNSCC immune cell types. The dot color reflects communication probabilities, and the dot size represents computed *P*-values. Empty space means the communication probability is zero. **d** Heatmap of CellChat analysis depicting dominant cell types involved in SPP1 signaling. **e** Violin plot showing expression of SPP1 receptor by cell type from the scRNA-seq data. **f** Schematic representation of the interaction mode of aberrant epithelial cells and immune cells in HNSCC

Among the cells identified in the atlas of HNSCC, the immune cell cluster formed the largest population. We extracted immune cells and subdivided them into eight subcategories: B/plasma, CD4 + T, CD8 + T, DC, macrophages, mast, monocytes, and Tregs cells (Fig. 10a, b and Supplementary Fig. S12a–c). We found that the receptor of PLAU, named PLAUR, was expressed in those immune cell clusters (especially in monocyte/macrophage clusters), suggesting that epithelial cells utilize intercellular signaling to drive immune cell recruitment via PLAU-PLAUR interaction in HNSCC (Fig. 10c and Supplementary Fig. 12d). The results of RT-qPCR and immunofluorescence in clinical tissue samples confirmed that PLAUR was 1.8 times more highly expressed in HNSCC tissue samples than HC and was mainly expressed in macrophages (Fig. 10d, e). Consistent with the predicted results, the mRNA and protein expression of PLAUR were higher in HNSCC patients, suggesting that high PLAU-PLAUR expression may mediate malignant cellular behaviors, leading to a worse clinical outcome in HNSCC patients (Supplementary Fig. S12e–g). Moreover, the results of the AUCell analysis found that multiple tumor-related signaling pathways were enriched within the expressed genes for monocytes and macrophages (Supplementary Fig. S13). Whereafter, CellChat was performed to analyze intercell communication within the immune cells and noted that macrophages/monocytes dominated the interaction with other immune cells (Fig. 11a, b). This intercell communication might be mediated mainly by SPP1 regulation (Fig. 11c, d). SPP1, also known as osteopontin, is upregulated in various cancers and involved in various biological processes, such as inflammation, fibrosis, cancer progression, and metastasis.^25^ Our result indicated that macrophages were the main source of SPP1 and various kinds of immune cells, including B cells, CD4 + T cells, CD8 + T cells, mast cells, and neutrophils, could serve as the receiver or the influencer of SPP1 (Fig. 11c, d and Supplementary Fig. S14a). SPP1 plays an important role among immune cells. The mRNA and protein expression of SPP1 were elevated in patients with HNSCC, and patients with high expression of SPP1 have a worse clinical prognosis (Supplementary Fig. S14e–g). Of all the SPP1 ligands, SPP1–CD44 interaction most commonly exists in immune cells, especially between macrophages and neutrophils/mast cells (Fig. 11e and Supplementary Fig. S14a–d). Collectively, these data suggest that the overexpressed PLAU in tumor epithelia could interact with its receptor PLAUR located on the surface of immune cells (especially macrophages), to influence the interaction mode of aberrant epithelial cells and immune cells in HNSCC (Fig. 11f).

In conclusion, this study confirms that Cuproptosis influences the malignant cell behavior and epithelial–immune interaction in HNSCC. We integrated scRNA-seq analysis of 6 HNSCC tissues and bulk genomic information of 502 HNSCC tissues to comprehensively assess the underlying cellular distribution of CRL and its correlation with major non-immune and immune features in HNSCC. The key CRLs could regulate the TME characteristics of HNSCC. Using a random forest (RF) model, we selected the key lncRNA JPX among these CRLs and implemented a ceRNA network. The upregulation of the JPX/miR-193b-3p/PLAU axis in malignant epithelial cells promoted cell proliferation, migration, and invasion in HNSCC. PLAU overexpressed in tumor epithelia could increase its binding with the receptor PLAUR, expressed mainly on macrophages, to ultimately influence the aberrant epithelial–immune interactome in HNSCC. We propose the combination of JPX and its downstream gene urokinase-type plasminogen activator (PLAU) inhibitors as a novel therapeutic strategy to target HNSCC and provide new insights into precision oncology (Supplementary Fig. S15).

## Discussion

In recent years, the incidence of HNSCC has risen worldwide, which accounts for more than 600 000 cases and 380 000 deaths annually, posing a tremendous threat to global human health.^2,26^ Hence, robust and clinically useful diagnosis biomarkers and effective therapeutic strategies are urgent needs for patients with HNSCC.^27^ Researchers have found that cuproptosis showed promising potential in the prognosis and treatment of tumors.^28^ The specific mechanism of cuproptosis is that copper ions induce protein toxic stress responses by binding to fatty acid acylated components in the tricarboxylic acid cycle (TCA) cycle, ultimately leading to cell death.^29^ Malignant transformation of head and neck neoplasia, can be related to dysregulation of TCA activity.^30^ Growing evidence suggests that the role of lncRNAs in cuproptosis cannot be ignored. In HNSCC, lncRNAs are inextricably entwined with malignant cell behaviors, cancer immunity, programmed cell death, and drug resistance, making them well-suited as relevant biosignatures and therapeutic targets.^31,32^ Nevertheless, the role and the specific mechanism of CRLs in HNSCC remain elusive.

As a CRL, JPX is involved in various types of cancers and is a biomarker that may indicate a poor prognosis of cancers.^33,34^ Being identified as an oncogenic regulator in lung cancer, JPX facilitates lung tumor growth and induces epithelial–mesenchymal transition (EMT) and lung cancer cell invasion by upregulating Twist1.^33^ The expression level of JPX is also upregulated in esophageal squamous cell carcinoma and is relevant to enhanced invasion and angiogenesis of esophageal squamous cell carcinoma.^35^ Herein, we found that JPX was also significantly upregulated in epithelial cells of HNSCC. To further explore the relationship between JPX and HNSCC, we used CAL27, a human malignant epithelial cell line, and found that the upregulation of JPX promoted CAL27 cell proliferation, migration, and invasion. Recently, ceRNA has become a concerned component of post-transcriptional regulators that influence tumor development by regulating the corresponding gene expression via miRNA-mediated mechanism.^36^ Mediating a new ceRNA regulatory network, overexpressed JPX in CAL27 cells can act as molecular sponges to competitively bind more miR-193-3p in our results. Moreover, overexpression of miR-193b-3p partly abrogated JPX-enhanced malignant behavior and oncogene PLAU upregulation in CAL27 cells. Results in clinical tissue samples drew a consistent conclusion. Collectively, our results demonstrated that JPX can competitively bind miR-193-3p, leading to less PLAU inhibition mediated by miR-193-3p, providing new insights into exploring potential molecular and regulatory mechanisms of JPX in HNSCC.

PLAU and its cell surface receptor PLAUR have already been proven to participate in multiple stages of tumorigenesis. Upregulation of PLAU in tumor tissues was associated with epithelial–mesenchymal transition, ECM degradation, cell proliferation, hypoxia, angiogenesis, stemness, and metastasis.^37–39^ However, there is a lack of research to date assessing the association between PLAU expression and immunomodulation of HNSCC. The immune system is now recognized to play a core role in tumor development.^40^ The effects of immuno-oncology therapies and targeted therapies are highly dependent on the understanding of the molecular mechanisms mediated by multifaceted interactions between malignant epithelial cells and immune cells within the TME. Nowadays, novel technologies such as scRNA-seq allow us to decipher complex intercellular interactions. In the single-cell map of our study, the overexpressed PLAU was mainly distributed in malignant epithelial cells of tumor tissue. Further pathway and enrichment analysis revealed that the overexpression of PLAU was directly relevant to immune regulatory processes as well as vital oncogenic pathways. Interestingly, the receptor of PLAU, PLAUR, is expressed mainly on macrophages. The Macrophage compartment was found to be the most significantly expanded in the immune cluster in HNSCC and has strong cell–cell communication with other immune cells by SPP1 signaling. Our findings supported that PLAU overexpressed in tumor tissue could interact with PLAUR to regulate the crosstalk between malignant epithelial cells and immune cells, presumably favoring immune escape in HNSCC.

The suitable therapy for each HNSCC patient is likely to depend on both individual clinical and molecular features of the tumor. Thereby, gaining insight into the molecular mechanism of HNSCC may enable researchers to identify patients who may benefit from certain therapeutic strategies. In our research, a correlation analysis between JPX and predicted IC50 drug data from the GDSC, CTRP, and PRISM databases was first performed. As a result, Tozasertib, Brivanib, and Axitinib were found to have the most relevant potential effects in the JPX-high HNSCC subclass. Till now, investigations on the therapeutic potential of Tozasertib and Brivanib in HNSCC are totally absent, demanding further studies and clinical evidence. In the current study, the researchers found that Axitinib had the potential to target epidermal growth factor receptor (EGFR) and was associated with improved survival in patients with heavily pretreated head and neck cancer.^41^ Subsequently, we performed a drug susceptibility analysis of PLAU and found that Dasatinib, Midostaurin, and Palbociclib had the most relevant potential effects in the PLAU-high HNSCC subclass. Dasatinib, a dual Src/Abl inhibitor, has been approved for the treatment of specific forms of leukemia.^42^ Previous research supported that dasatinib might represent a treatment option for patients suffering from Discoidin domain-containing receptor 2 (DDR2) -high HNSCC subclass.^43^ Midostaurin is a multi-kinase inhibitor initially developed for treating patients with other solid malignancies and has been identified as a sensitive drug for SPARC/MMP9/CD44 in HNSCC recently.^44^ Palbociclib is the first cyclin-dependent kinase (CDK4/6) inhibitor approved by the FDA and has been confirmed for its effectiveness in HNSCC second-line treatments. Significant synergistic effects were observed in rational combinations with palbociclib of multiple agents, including inhibitors of the PI3K, EGFR, and MEK pathways.^45,46^ Previous studies have reported that EGFR inhibitor and cyclin D1-CDK4/6 inhibitor may potentially synergize to impede tumor growth and progression via metabolic disruption, ROS scavenging, and cell cycle arrest in HNSCC preclinical models.^47^ Meanwhile, based on our results, EGFR inhibitor Axitinib and cyclin D1-CDK4/6 inhibitor Palbociclib in-combination therapeutic strategy is capable of synergistically targeting JPX/miR-193b-3p/PLAU axis, which may reduce the cell malignant behavior and influence tumor immunity to further enhance HNSCC suppression. Despite the requirement of further preclinical and clinical trials, this novel combined therapeutic strategy provides the potential to revolutionize the current HNSCC treatment.

In summary, we revealed the crucial role of JPX (the key CRL) in the processes in HNSCC. We constructed a JPX-mediated ceRNA network using bioinformatics tools and identified that the JPX/miR-193b-3p/PLAU axis contributed to the aberrant behavior of malignant epithelial cells via scRNA-seq analysis. This conclusion was confirmed by further experiments of CAL27 cells and outcome data of clinical samples. Meanwhile, we found that PLAU, the crucial downstream gene of JPX, was also capable of regulating the aberrant epithelial–immune interaction in HNSCC by binding to its receptor PLAUR on immune cells. Eventually, we propose the combined application of JPX inhibitor Axitinib and PLAU inhibitor Palbociclib as a novel potential therapeutic strategy for the targeted treatment of HNSCC, which was also consistent with the HNSCC clinical therapeutic recommendation as the combination of EGFR and CDK4/6 inhibitors. This finding might provide a robust theoretical clue for clinical combination therapy in HNSCC patients. Nevertheless, our current study still has certain limitations. Clinical samples used in this work were at a single time point which may rarely reflect the impact of spatial and temporal intratumoral heterogeneity. Additionally, how the novel potential therapeutic strategy we have proposed affected the tumorigenesis of HNSCC via the JPX/miR-193b-3p/PLAU signaling axis should be further explored.

Moving forward, our research will focus on investigating cuproptosis-related biomarkers through the integration of machine learning and multi-omics methodologies. Moreover, our research aims to integrate targeted therapeutics focused on the JPX/miR-193b-3p/PLAU axis with nanomaterials to enhance precise and controlled drug release, rapid response to external triggers, and segmented drug administration. These advancements show potential for enhancing personalized and targeted therapeutic approaches. Additionally, we will explore the synergistic impacts of combining targeted medications with radiation and chemotherapy. These investigative paths offer promise for introducing innovative and progressive strategies in the treatment of HNSCC.

## Materials and methods

### scRNA sequencing data processing

The scRNA dataset GSE172577 and GSE164241 were extracted from the GEO (https://www.ncbi.nlm.nih.gov/) database. RNA-seq data and sample annotation information were obtained using the Seurat R package (version 4.0.2). Cells with <200 genes, >5 500 genes, >10% mitochondrial genes, or >3% hemoglobin content were filtered out. After normalizing scRNA-seq data and controlling for the relationship between average expression and dispersion, highly variable genes were identified in each cell. Differentially expressed genes (DEGs) for a given cell type were determined with the FindAllMarkers function, and a UMAP was used to dimension reduction and classify the cells into different clusters.

### Cell–cell communications analysis

To explore the crosstalk pattern between cells, we employed the R package CellChat. Briefly, we followed the official workflow and created the CellChat object based on a normalized count matrix. We performed permutation tests to evaluate the statistical significance and used default on method parameters.

### Data collection

RNA-sequencing (RNA-seq) expression profiles of 501 HNSCC tissues and 44 adjacent normal tissues were extracted from The Cancer Genome Atlas (TCGA) database (https://portal.gdc.cancer.gov/), and the corresponding clinical data was obtained from cBioPortal Database Profiles.

### Identification of CRLs

A total of 10 cuproptosis-related genes were obtained, and Spearson correlation analysis was performed among HNSCC patients to identify CRLs with the “limma” package, and 146 lncRNAs with *R* > 0.4 and FDR < 0.001 were selected.

### Survival, differential analyses of CRLs

Univariate Cox regression analysis was performed to identify prognostic CRLs based on the expression data and Overall Survival (OS) time of 501 patients. Then, the Wilcoxon signed-rank test and “limma” package was utilized to evaluate the differential expression of these prognostic CRLs in HNSCC and normal tissues (|log2(FC)| > 0, FDR < 0.05). Moreover, the specific cuproptosis-related gene-to-lncRNA correlations were presented using Spearson correlation analysis (*P* < 0.05).

### Unsupervised cluster analysis based on CRLs to identify HNSCC subtypes

The unsupervised cluster analysis was utilized to classify the 501 HNSCC cases based on the 19 CRLs using the “ConsensusClusterPlus” package. The number of HNSCC subtypes and stability were determined by the Gap statistic and Elbow method. Kaplan–Meier (K-M) Survival curve was used to compare the OS to evaluate the relationship between prognosis and each subtype. Moreover, the immune features and TME characteristics among the distant CRLs-based subtypes were compared using the Wilcoxon signed-rank test, which covered the aspects of enrichment levels of immune infiltration cells and pathways in the tumor samples.

### Gene set enrichment analysis

A total of 158 DEGs were obtained by differential analysis of gene expression profiles of high- and low-risk patients in the TCGA-HNSCC cohort following the standards of |log_2_ (FC) | > 0.5, FDR < 0.05. We performed the gene set enrichment analysis (GSEA), including gene ontology (GO), and Kyoto encyclopedia of genes and genomes (KEGG) analyses to further understand the potential mechanisms behind the DEGs. FDR < 0.05 was considered statistically significant.

### Construction of the least absolute shrinkage and selection operator (LASSO) model and support vector machine-recursive feature elimination (SVM-RFE) feature selection process

The LASSO regression and the SVM algorithm were used to screen the diagnostic markers of HNSCC. To implement LASSO, the “glmnet” package was used, the binomial response type was selected, and the alpha was set to 1. Furthermore, the SVM-RFE can find the best variables as a surveillant machine learning method to support vectors by cutting the features in half each round if there are many features. The SVM classifier from the R package (http://github.com/johncolby/SVM-RFE) was adopted to classify the selected biomarkers in the diagnosis of HNSCC. Validation proceeded with 10-fold cross-validation. The features were cut in half each round until there were less than 100 remaining.

### CeRNA network construction and functional annotation

First, we identified the target miRNAs located downstream of 11 CRLs in the miRbase database. Meanwhile, differentially expressed miRNAs (DEmiRNAs) were obtained based on |log_2_(FC) | > 0.5 and FDR < 0.05 using the Wilcoxon signed-rank test in 501 HNSCC and 44 normal tissues, and the target DEmiRNAs were identified for further study. Second, based on these DEmiRNAs, candidate target mRNAs were found in the miRTarBase, miRDB, and TargetScan databases. Subsequently, differentially expressed mRNAs (DEmRNAs) were identified according to the same criteria, and the final target DEmRNAs were determined by marching DEmRNAs to the candidate target mRNAs obtained in the above three databases. Finally, a novel lncRNA-miRNA-mRNA regulatory ceRNA network was presented by Cytoscape.^25^ In addition, GO and KEGG enrichment analyses were implemented to explore the underlying functions and related pathways of the ceRNA network.

### Drug sensitivity analysis

Chemotherapeutic response in HNSCC patients was assessed utilizing the genomics of drug sensitivity in cancer (GDSC) database (https://www.cancerrxgene.org), the cancer therapeutics response portal (CTRP) database (https://portals.broadinstitute.org/ctrp.v2.1/) and the profiling of relative inhibition simultaneously in mixtures (PRISM). “Ggplot2” R package was used to explore the correlation between the JPX/PLAU expression and IC50 of anti-tumor drugs by heat maps and point diagrams.

### Spatial transcriptomics analysis

The spatial transcriptome sequencing dataset GSE181300 was extracted from the GEO (https://www.ncbi.nlm.nih.gov/) database. Sample GSM5494482 and GSM5494481 were used for subsequent analysis. Spatial transcriptomics data analysis was performed using the “Seurat” R package, employing methods similar to those used for scRNA-seq data analysis. The “FindTransferAnchors” and “TransferData” functions, with default settings from the “Seurat” R package, were utilized to predict single-cell data on the spatial transcriptomics data.

### CAL27 cell culture

Human tongue squamous carcinoma cell line (CAL27) was cultured in high glucose Dulbecco’s modified Eagle’s medium (DMEM) supplemented with 10% fetal bovine serum (Gibco, USA) and 1% penicillin−streptomycin formulation (Gibco, USA) at 37 °C with 5% CO_2_. For JPX knockdown, CAL27 cells were transfected with siRNA segments targeting JPX or negative/positive control (Yibaike, Beijing, China) for 18–48 h using Lipofectamine 3000 (Invitrogen, USA). Stable JPX overexpression in the CAL27 cell lines was achieved by transduction with lentivirus encoding (pCDH-CMV-JPX-EF1-CoGFP-T2A-puro, designed and synthesized by Yibaike (Beijing, China)). Empty vectors were used as the negative control. siRNA against miR-193b-3p and miR-193b-3p mimic (Yibaike, Beijing, China) were transfected into CAL27 cells using Lipofectamine 3000 (Invitrogen, USA) according to the manufacturer’s instructions. Cells transfected with NC plasmid were identified as control groups. The transfection efficiencies were validated by qPCR analysis.

### Cytotoxicity assays

After seeding CAL27 in 24-well plates for 1, 2, 3, or 4 days, a Cell Counting Kit-8 (CCK-8, Beyotime, China) was applied to evaluate cell proliferative ability. The CCK-8 solution was diluted with a medium at a ratio of 1:10 and then incubated with cells in a 37 °C incubator for 2 h. Subsequently, the medium was collected from each well for further OD value measurement at 450 nm. In addition, the cytotoxicity of different treatments was also measured using a Calcein-AM/PI Viability/Cytotoxicity Kit (Solarbio, Beijing) according to the protocol provided by the manufacturer.

### Transwell migration assay

CAL27 cells were suspended in serum-free DMEM and transferred to the upper chamber of a transwell with 8 μm pores (Costar, Cambridge, MA, USA). The bottom chamber of the Transwell was filled with cell culture medium with 10% FBS to drive cell migration for 48 h. The upper surface of the upper chamber was cleaned after 24 h, and the bottom surface of the upper chamber was stained with 0.1% crystal violet. Images were captured using a microscope.

### Wound healing assay

CAL27 cells were suspended in a cell culture medium at a density of 2 × 10^4^ cells per mL and transferred to a mold chamber (ibidi, Germany) with a 1 mm wide insert. Insert was removed after the cell reached> 95% confluent to allow cell migration. Cleaned areas were captured using a microscope from Day 0 to Day 2 and measured using ImageJ software (ImageJ, RRID:SCR_003070).

### Patients and samples

We collected 12 HNSCC tumor samples at the Stomatology Hospital, Zhejiang University School of Medicine. All participants signed informed consent. All samples were collected with the approval from the Stomatology Hospital of Zhejiang University School of Medicine Human Research Ethics Committee (Ethics number: I20230936).

### Ribonucleic acid (RNA) isolation and quantitative reverse-transcription polymerase chain reaction (RT-qPCR)

Total RNA samples were isolated from CAL27 cells using TRIzol reagent (Ambion, Inc., Austin, Texas, US) following the manufacturer’s protocol. We reverse-transcribed 1 μg template RNA using a PrimeScript RT reagent kit (TaKaRa Bio, Shiga, Japan) and measured expression of the target gene using an SYBR Fast Universal qPCR Kit (Kapa Biosystems, Inc. [Roche Life Science, Basel, Switzerland]) for real-time qPCR. Primer sequences are provided in Table S2. We normalized the final calculated results to GAPDH and converted them using relative quantification (2^-ΔΔCt^).

### Lipid ROS measurement

Lipid ROS level was analyzed by immunofluorescence using DCFH-DA (Sigma, USA) fluorescent probe. The sections were incubated with DCFH-DA fluorescent probe for 1 h at room temperature in the dark. Nuclei were counterstained with 4’, 6-diamidino-2-phenylindole (DAPI). Confocal microscopy (Nikon) was used for imaging.

### Immunohistochemistry

Formalin-fixed paraffin sections of HNSCC tissues were subjected to rehydration and antigen retrieval. Next, we blocked the sections with bovine serum albumin (BSA) and incubated them with primary anti-PLAU (Abcam, Cambridge, UK) per the manufacturer’s protocol. Slides were incubated with HRP-conjugated secondary antibody (Abcam, Cambridge, UK), visualized using the 3,3’-Diaminobenzidine (DAB) technique, counterstained with hematoxylin and dehydrated. Sections were evaluated under an optical microscope. Antibody catalog number: anti-PLAU (Abcam, Cambridge, UK, ab24121).

### Immunofluorescence

We blocked paraffin sections with BSA and then incubated them with primary antibodies (anti-LIAS, anti-FDX1, anti-CD68, anti-PLAU, anti-PLAUR, or anti-Ki67) overnight at 4 °C in a humidified chamber. Slides were then incubated with fluorescein-conjugated secondary antibodies for 1 h at room temperature in the dark. Nuclei were counterstained with 4’, 6-diamidino-2-phenylindole (DAPI). All antibodies involved in this experiment were purchased from Abcam. We used confocal microscopy (Nikon) for imaging. Antibody catalog number: anti-PLAU (Abcam, Cambridge, UK, ab24121), anti-LIAS (Abcam, Cambridge, UK, ab246917), anti-FDX1 (Abcam, Cambridge, UK, ab109312), anti-CD68 (Abcam, Cambridge, UK, ab955), anti-PLAUR (Cell Signaling Technology, Boston, USA, #12713), anti-Ki67 (Abcam, Cambridge, UK, ab16667).

### PLAU single-gene bioinformatic analysis and construction of the ceRNA network

Based on the median expression value of the key gene PLAU, HNSCC patients were divided into high- and low- expression groups. Genes with |log_2_FC | > 1 and FDR < 0.05 were identified as DEGs using the R package “limma”, and these DEGs were then enrolled in GO and KEGG enrichment analysis. Biological pathways were compared between the high- and low-expression subgroups using GSEA software (http://www.broad.mit.edu/GSEA).

### Statistical analysis

Statistical analysis and graphs were generated using R software V4.0.2, SPSS software V26.0, and GraphPad Prism 8.0. Wilcoxon signed-rank test was used for subgroup analysis. Spearson correlation analysis and LASSO regression were utilized to screen target LncRNAs. Univariate and multivariate Cox regression analyses were utilized to evaluate prognostic significance. Two-sided *P* < 0.05 was used as the criterion for a statistically significant difference.

## Supplementary information

Supplemental material

## Data Availability

All the data associated with this study are available in the article and its Supplementary Information files and from the corresponding author upon reasonable request.
