# Supplementary material for: Cuproptosis-related lncRNA JPX regulates malignant cell behavior and epithelial-immune interaction in head and neck squamous cell carcinoma via miR-193b-3p/PLAU axis
Source: Int J Oral Sci. 2024 Nov 8;16:63. doi: 10.1038/s41368-024-00314-y (PMC11543849; doi:10.1038/s41368-024-00314-y)
Supplement: Supplementary file 1 — Supplemental material [file 41368_2024_314_MOESM1_ESM.pdf]

Cuproptosis-related lncRNA JPX regulates malignant cell behavior and epithelial-immune interaction in head and neck squamous cell carcinoma via miR-193b-3p/PLAU axis

Mouyuan Sun [1], Ning Zhan [1], Zhan Yang [1], Xiaoting Zhang [1], Jingyu Zhang [1], Lianjie Peng [1], Yaxian Luo [1], Lining Lin [1], Yiting Lou [1], Dongqi You [1], Tao Qiu [1], Zhichao Liu [1], Qianting Wang [1], Yu Liu* [1], Ping Sun* [1], Mengfei Yu* [1], Huiming Wang [1]

M. Sun, N. Zhan, Z. Yang, X. Zhang, J. Zhang, L. Lin, Y. Lou, L. Peng, D. You, T. Qiu, Z. Liu, Q. Wang, Y. Liu, P. Sun, M. Yu, H. Wang

[1] Stomatology Hospital, School of Stomatology, Zhejiang University School of Medicine, Zhejiang Provincial Clinical Research Center for Oral Diseases, Key Laboratory of Oral Biomedical Research of Zhejiang Province, Cancer Center of Zhejiang University, Engineering Research Center of Oral Biomaterials and Devices of Zhejiang Province, Hangzhou 310000

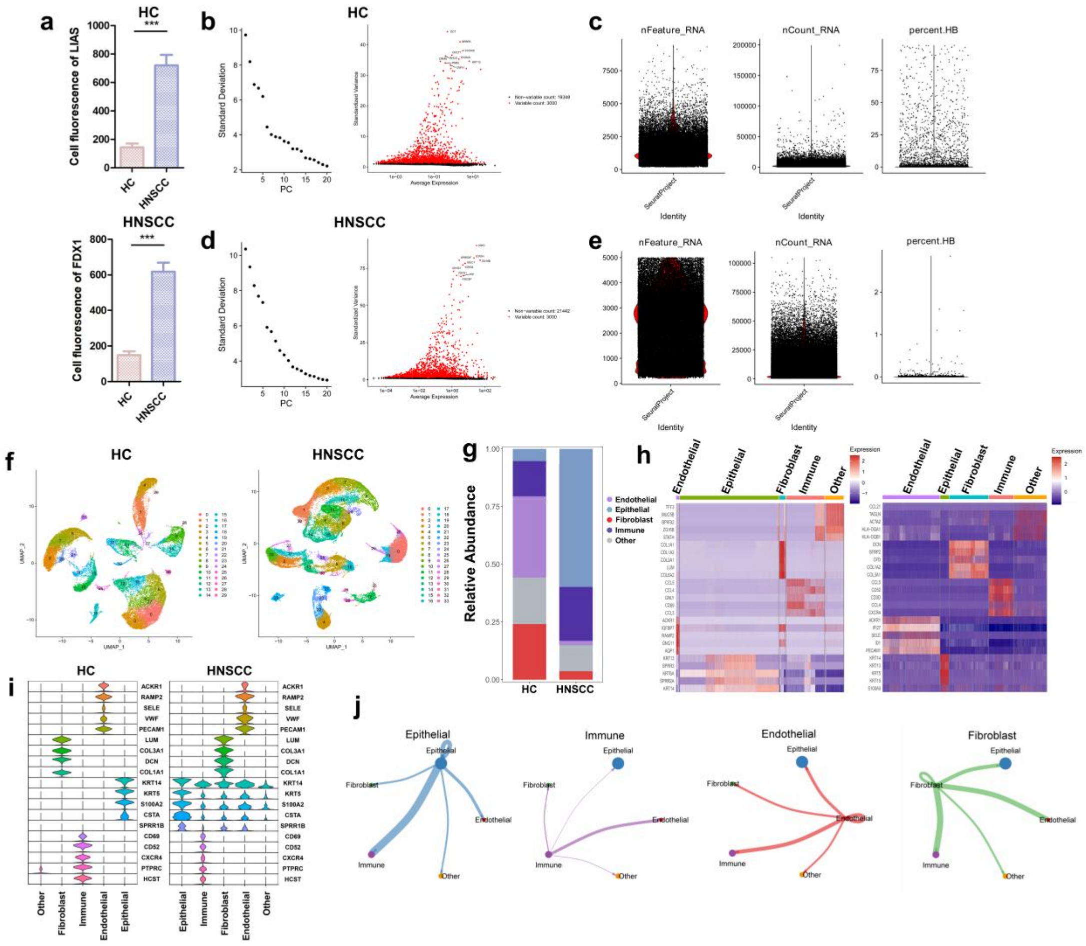

Figure S1. Supplementary information for the first-dimensional reduction and clustering. (a) Quantitative analysis of Immunofluorescence for the localization and the expression of LIAS and FDX1 in HNSCC clinical samples. (b-e) Included single-cell datasets before and after data cleaning. (f) UMAP dimensionality reduction of the total cells. (g) Bar graph of relative cell proportions. (h) Heat map for the top 5 DEGs of major cell populations. (i) Marker genes expressed in 4 major cell compartments. (j) Strength of ligand-receptor interactions between cell population pairs of HNSCC based on CellChat analysis. *$P < 0.05$, **$P < 0.01$, and ***$P < 0.001$.

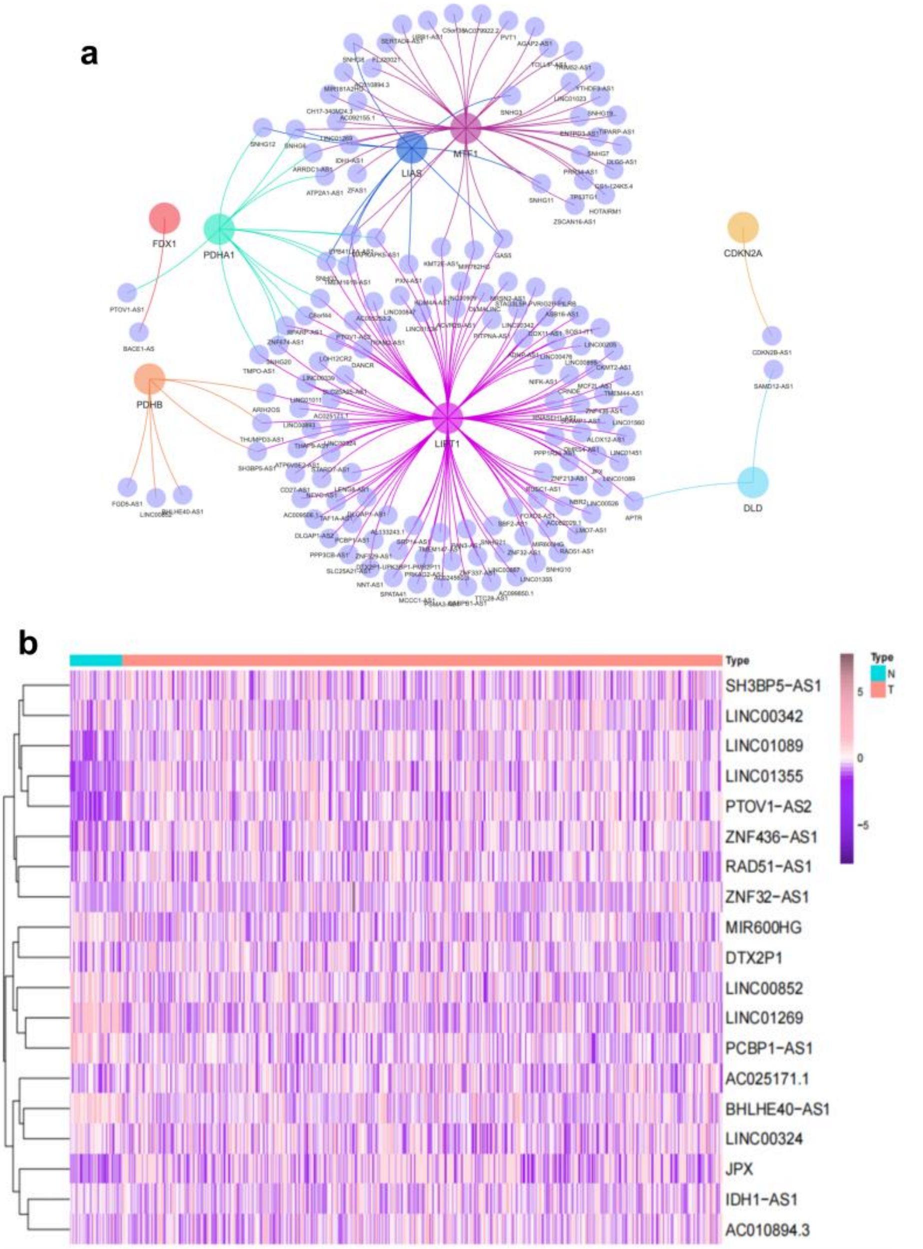

Figure S2. A co-expression network of cuproptosis-related genes (CRGs) and cuproptosis-related lncRNAs (CRLs). (a) A co-expression network of CRGs and CRLs. (b) Hierarchical clustering for expression of the 11 differentially expressed CRLs.

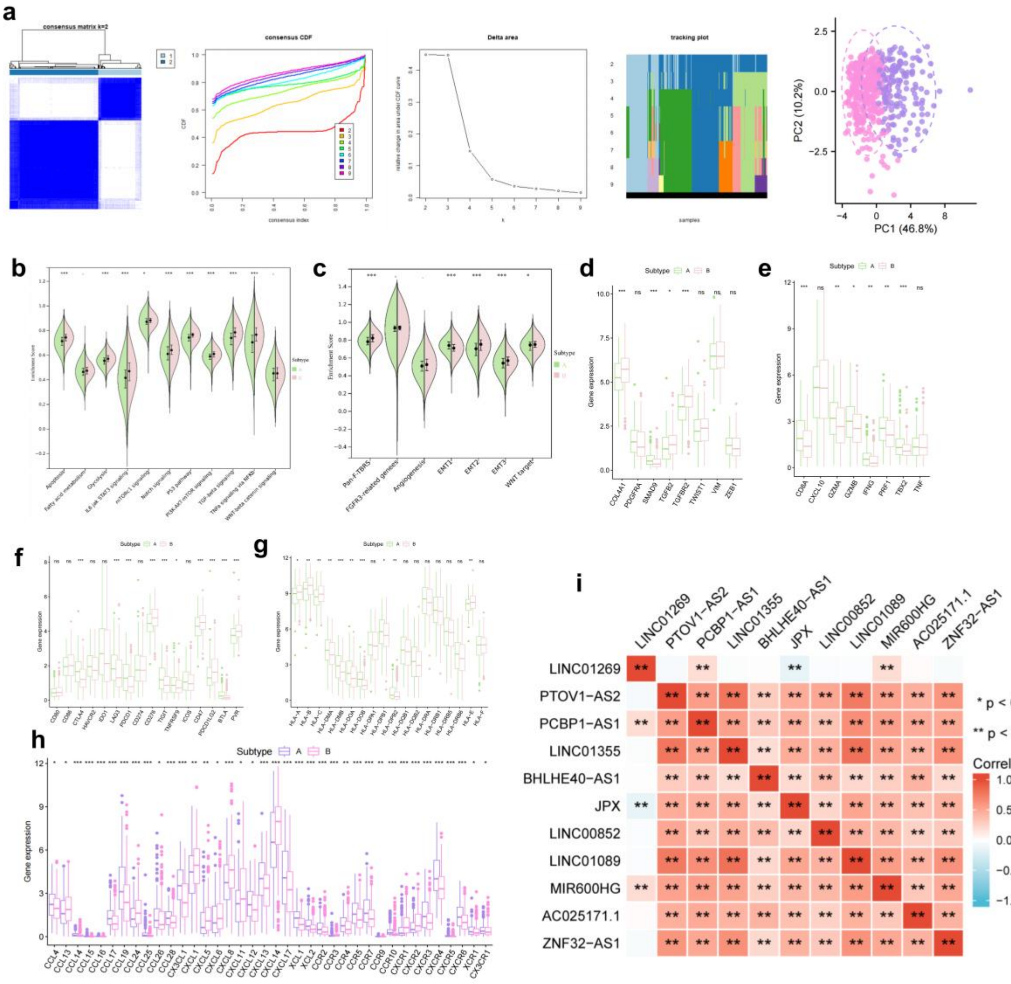

Figure S3. Unsupervised learning for cuproptosis lncRNA classification. (a) Unsupervised clustering of 11 CRLs and the optimized consensus matrix with k = 2. (b-c) Differentially expressed extracellular matrix-related pathways in Subtype A and Subtype B. (d-f) Different immune cell landscape in Subtype A and Subtype B. (g) Differentially expressed MHC-II-epitope complexes in Subtype A and Subtype B. (h) Enrichment levels of chemokines in 2 CRL clusters. (i) Spearman's correlation coefficient analysis of the 11 CRLs. $^{*}P < 0.05$, $^{**}P < 0.01$, and $^{***}P < 0.001$.

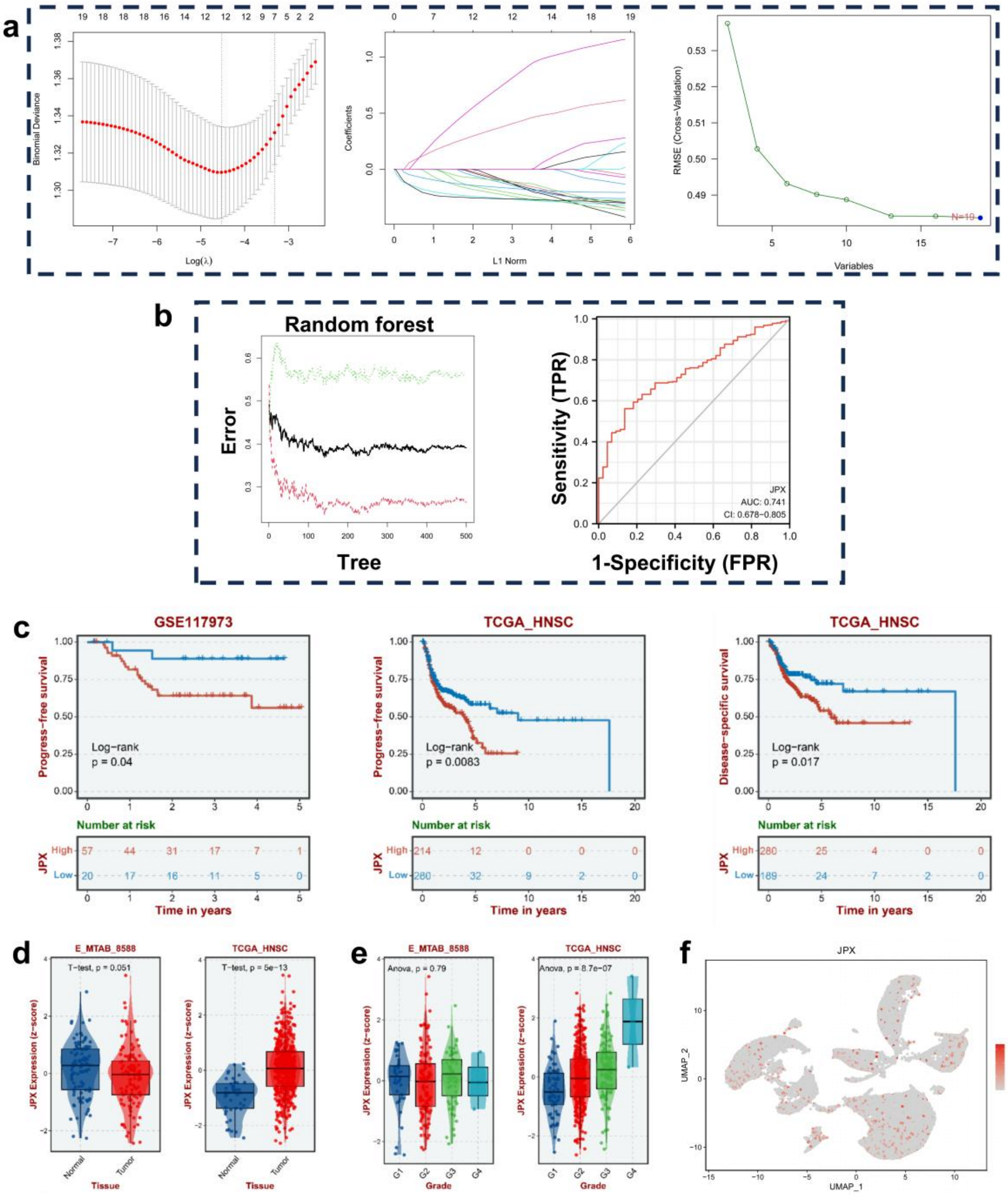

Figure S4. Identification of CRLs in HNSCC patients. (a) CRLs screened by the LASSO Cox regression model. (b) Random survival forest analysis of CRLs. (c) Kaplan-Meier analytical evaluation of the prognostic values of the candidate CRL JPX. (d) Prognostic values of JPX in the external test set and the training set from TCGA – HNSCC cohort. (e) The expression levels of JPX with Grade. (f) Expression of JPX is shown in UMAP of HC.

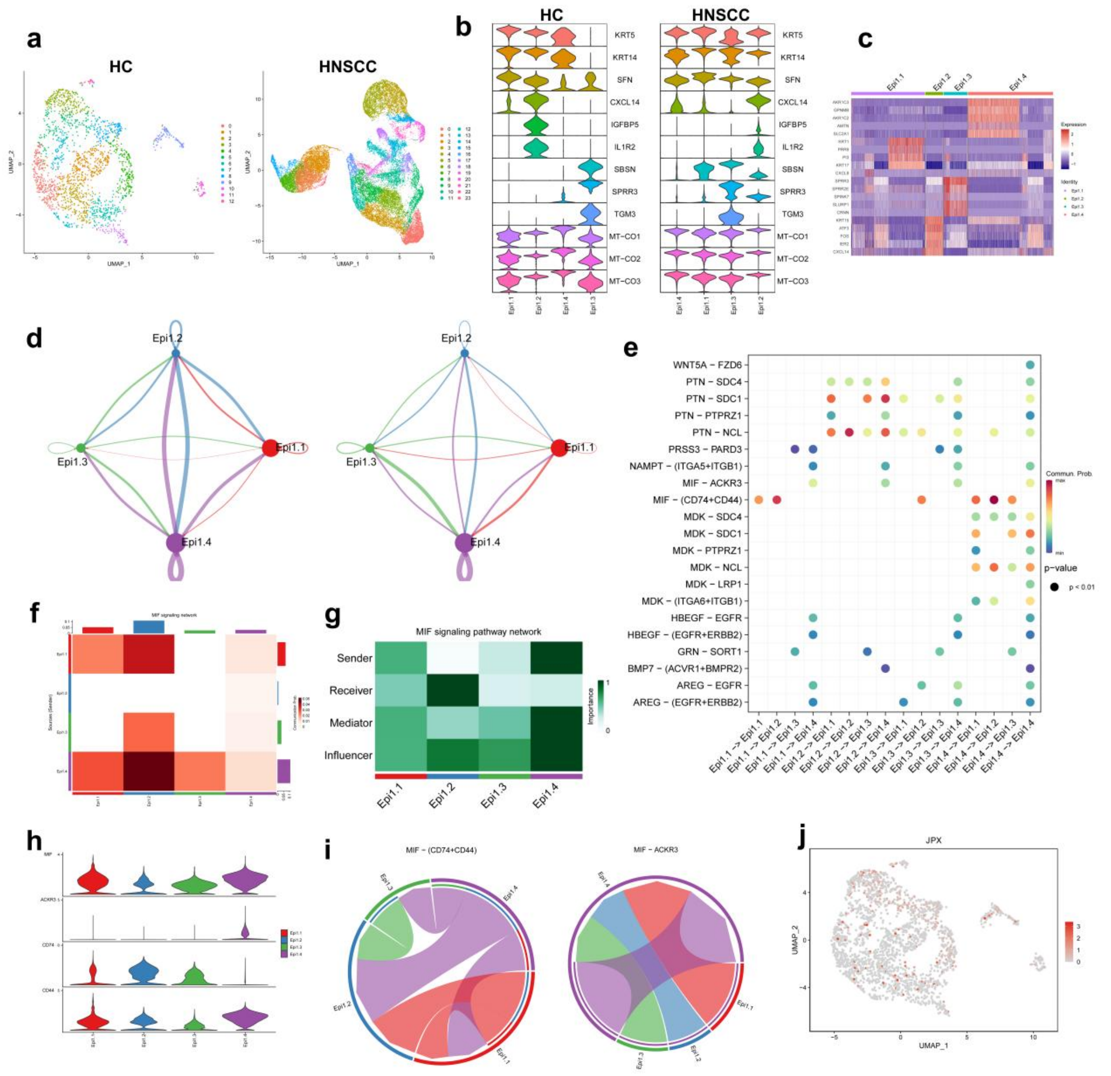

Figure S5. Single-cell transcriptome characterization of epithelial cells in HNSCC and the role of JPX in epithelial cells. (a) UMAP dimensionality reduction of epithelial cells. (b) Marker genes expressed in epithelial cell populations. (c) Heat map for the top 5 DEGs of epithelial cell populations. (d) Strength and number of ligand-receptor interactions between epithelial cell population pairs of HNSCC based on CellChat analysis. (e) Bubble plots of the significant differentially expressed ligand-receptor pairs of HNSCC epithelial cell types. (f) Heatmap of CellChat analysis depicting dominant cell types involved in MIF signaling. (g) Heatmap showing communication probabilities from senders (secretors) to receivers (targets)

for MIF signaling pathway. (h) Violin plot showing expression of MIF receptor by cell type from the scRNA-seq data. (i) Chord diagrams of the MIF-(CD74+CD44) and MIF-ACKR3 signaling network of HNSCC epithelial cell types. (j) Expression of JPX is shown in UMAP of epithelial cell populations in HC.

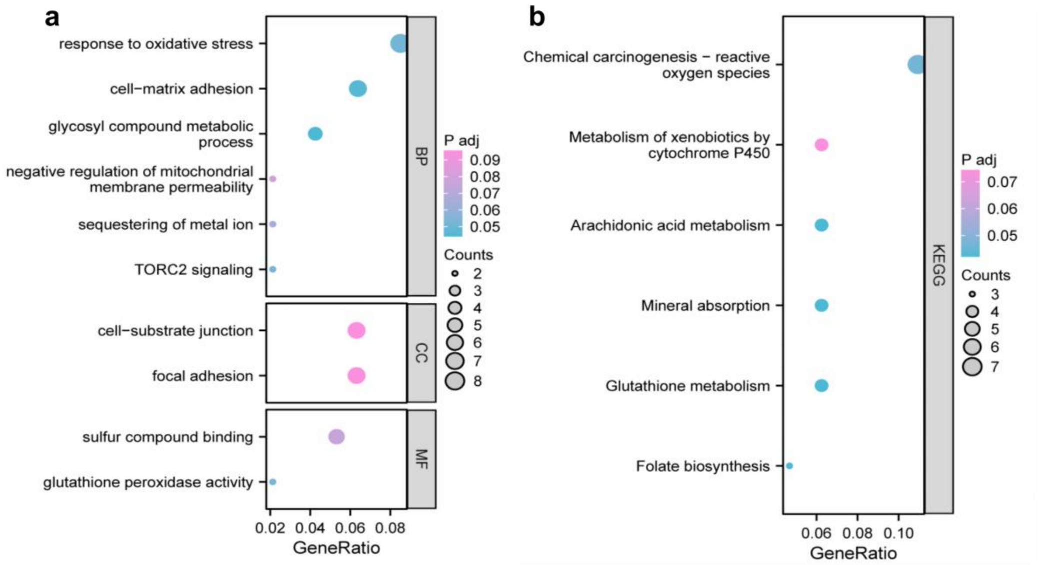

Figure S6. Enrichment analysis of malignant epithelial cells. (a) Dot plots of GO enrichments of malignant epithelial cells in HNSCC. (b) Dot plots of KEGG pathways of malignant epithelial cells in HNSCC.

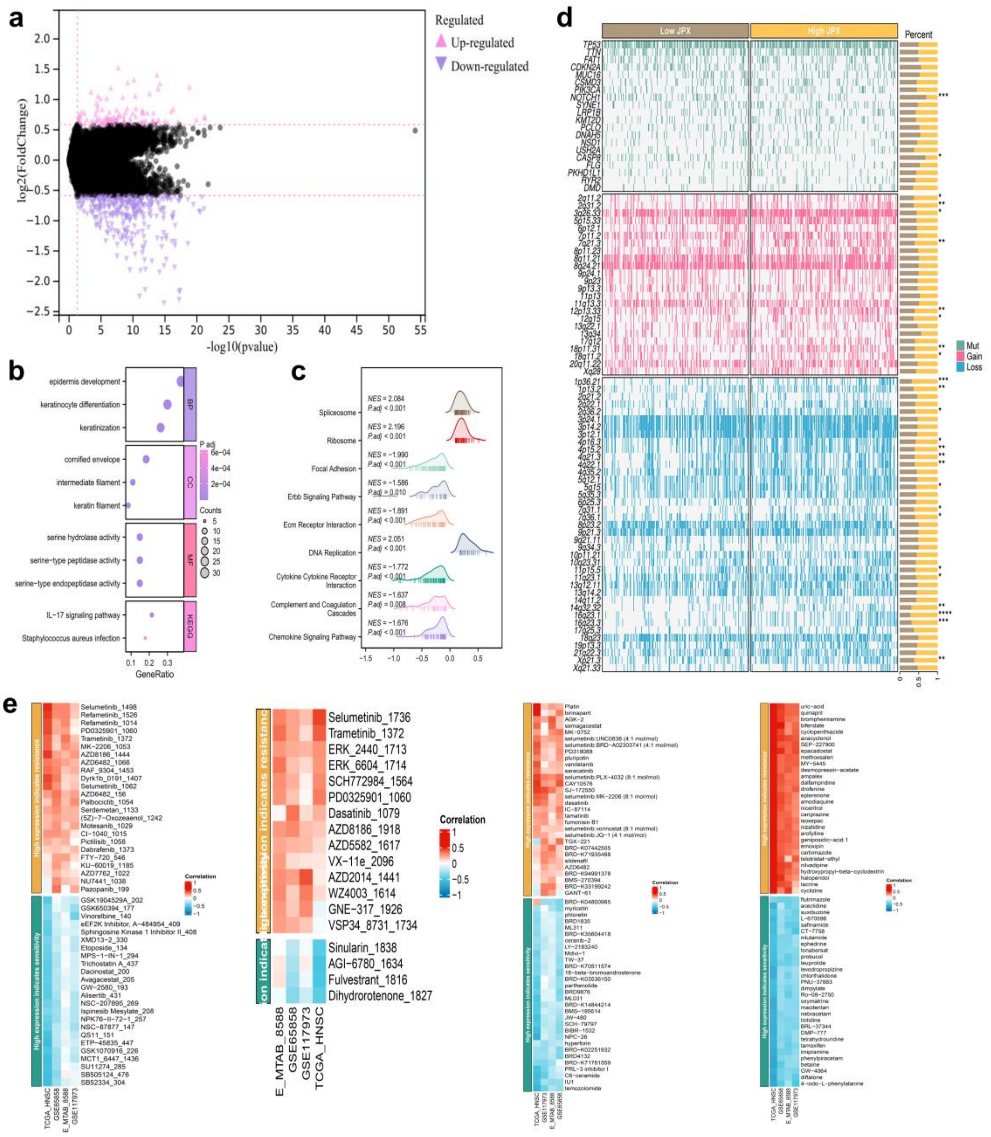

Figure S7. Functional analysis of JPX. (a) Volcano map of JPX-related differentially expressed genes. (b) Dot plots of GO enrichments and KEGG pathways associated with JPX in HNSCC. (c) GSEA analysis of JPX in HNSCC. (d) Somatic mutation characteristics of JPX high-expression and JPX low-expression subgroups in HNSCC. (e) Drug sensitivity analysis of JPX high-expression and JPX low-expression subgroups.

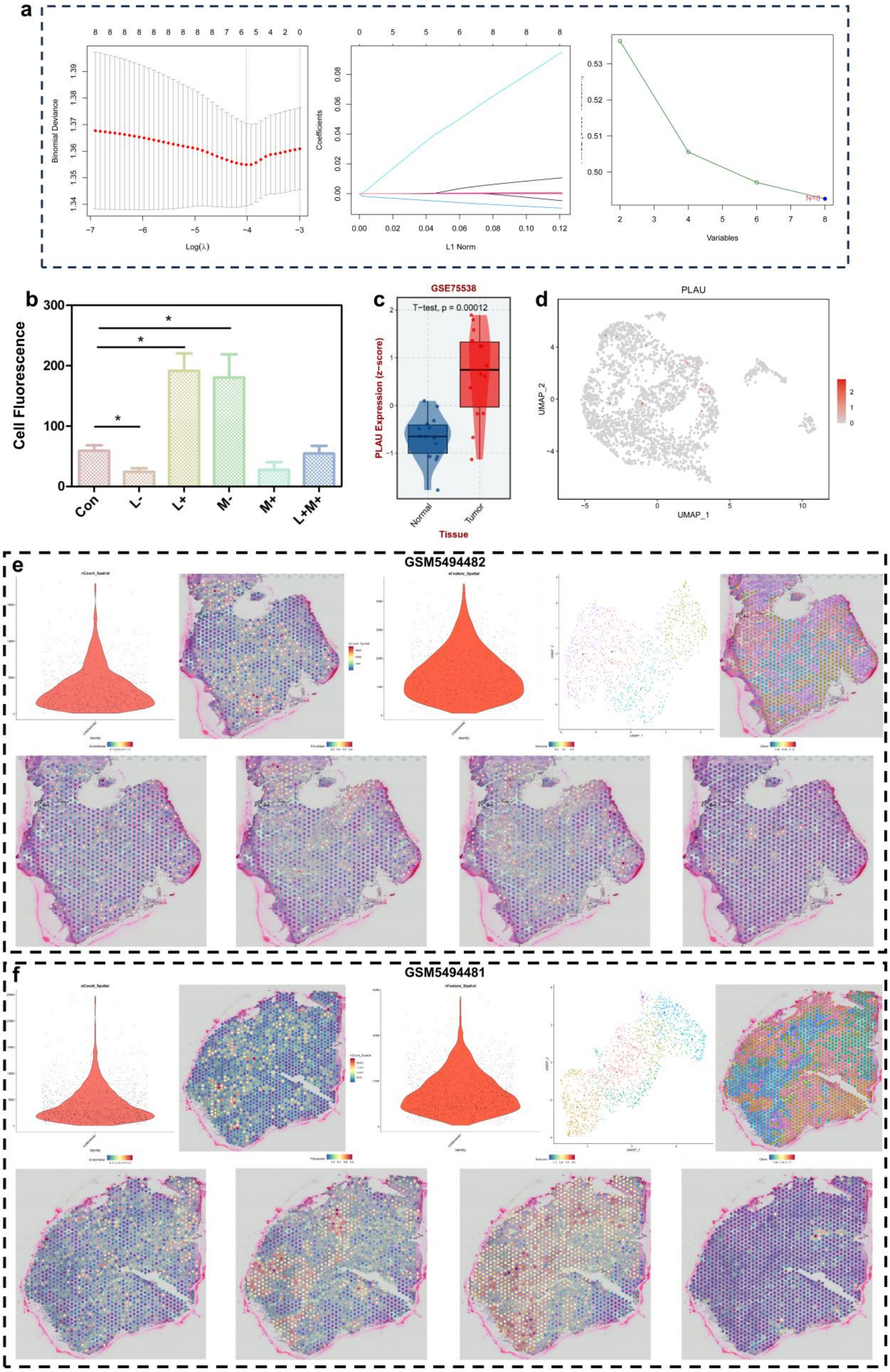

Figure S8. Expression levels of PLAU were explored using multi-omics. (a)

JPX-related miRNAs screened by the LASSO Cox regression model. (b) Quantitative analysis of Immunofluorescence for the localization and the expression of PLAU in CAL27 cells with JPX/miR-193b-3p knockdown or overexpression. (c) The expression level of PLAU between HC and HNSCC group. (d) Expression of PLAU is shown in UMAP of epithelial cell populations in HC. (e-f) Expression of endothelial cells, immune cells, fibroblast cells and other cells are shown in Spatial transcriptome data. *$P < 0.05$, **$P < 0.01$, and ***$P < 0.001$.

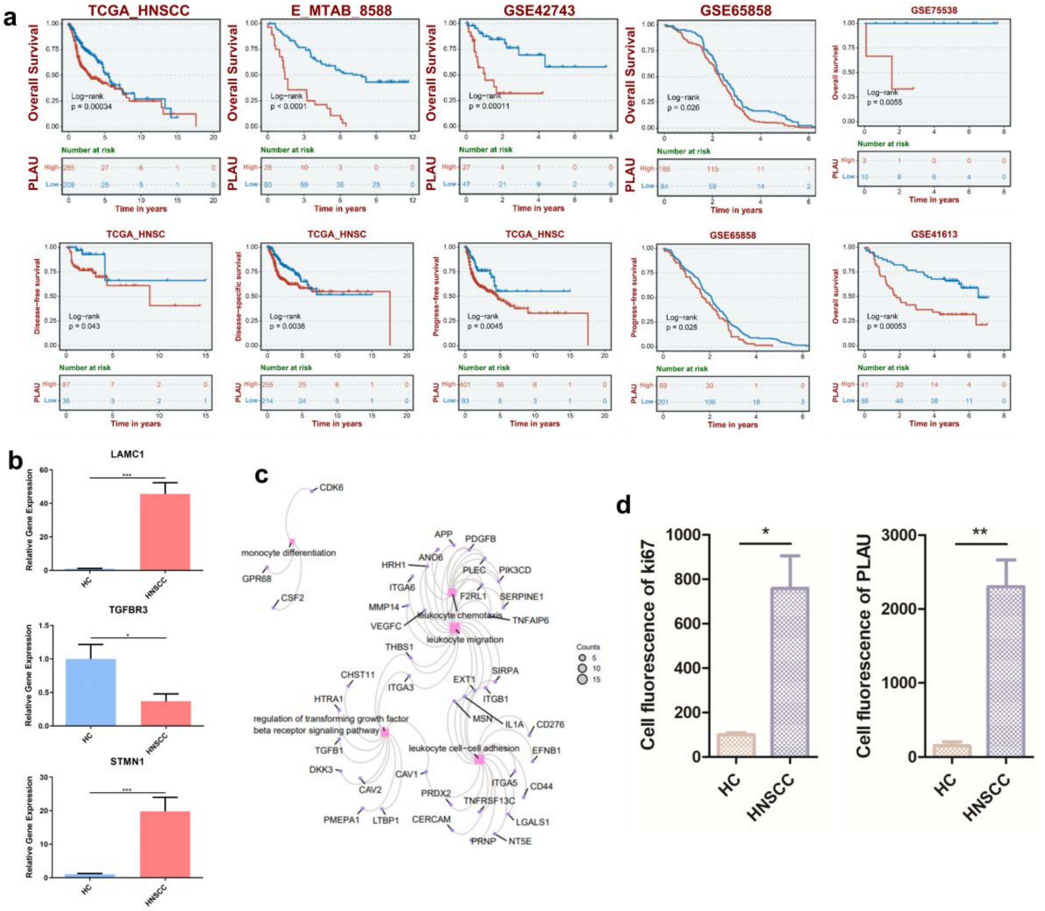

Figure S9. Clinical prognostic values of PLAU. (a) Kaplan-Meier analytical evaluation of the prognostic values of PLAU in the TCGA–HNSCC cohort and the external test set. (b) The relative expression levels of LAMC1, STMN1, and TGFBR3

in HNSCC clinical samples. n = 7 per group. (c) Interaction network of downstream of PLAU pathways and related key genes. (d) Quantitative analysis of Immunofluorescence for the localization and the expression of Ki-67 and PLAU in HNSCC clinical samples. *$P < 0.05$, **$P < 0.01$, and ***$P < 0.001$.

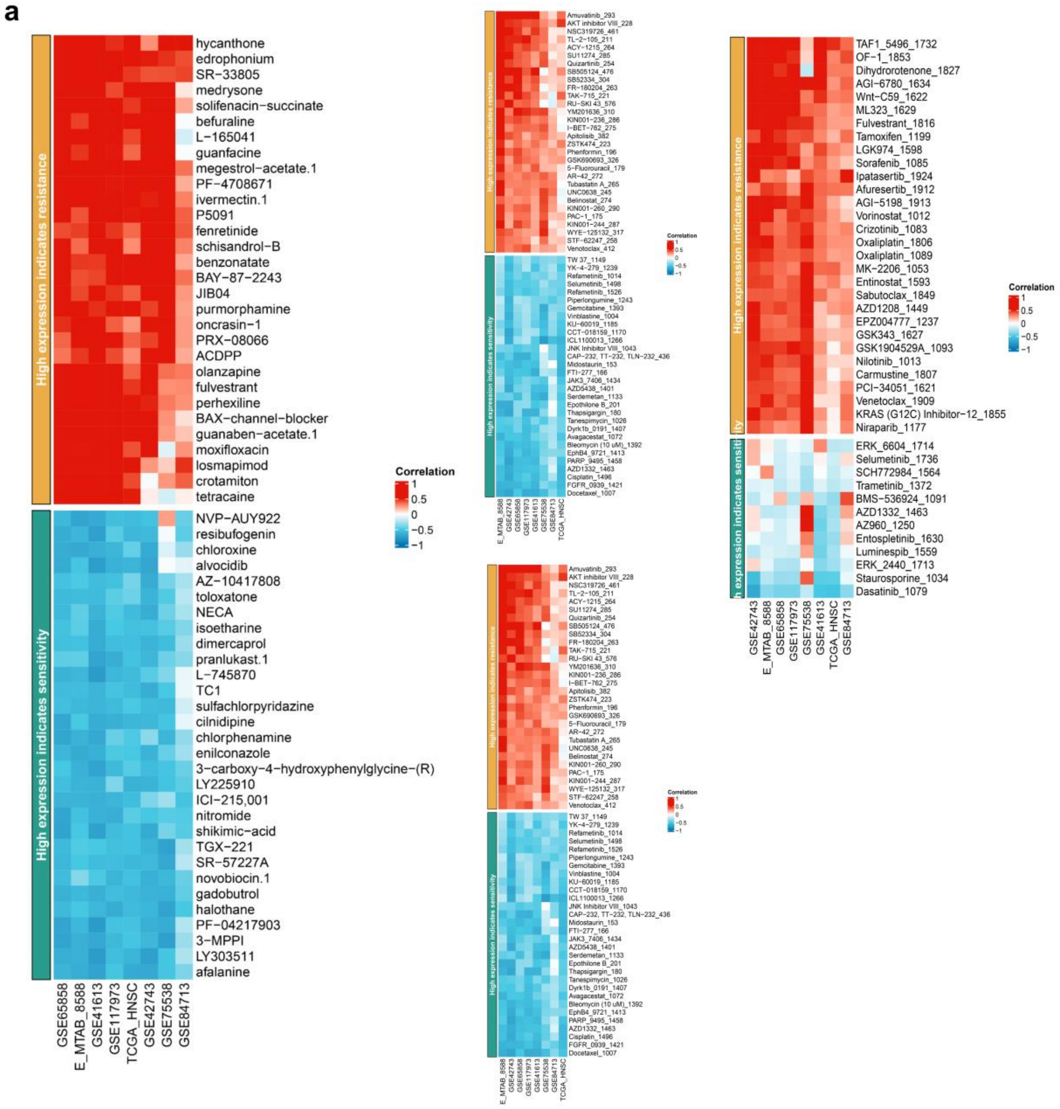

Figure S10. Drug sensitivity analysis of PLAU. (a) Drug sensitivity analysis of PLAU high-expression and PLAU low-expression subgroups.

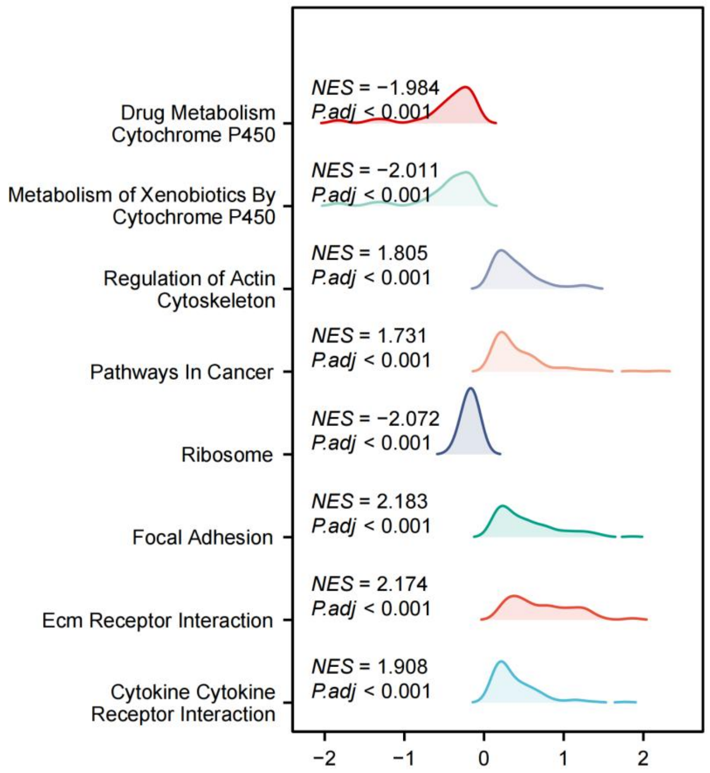

Figure S11. GSEA analysis of PLAU in HNSCC.

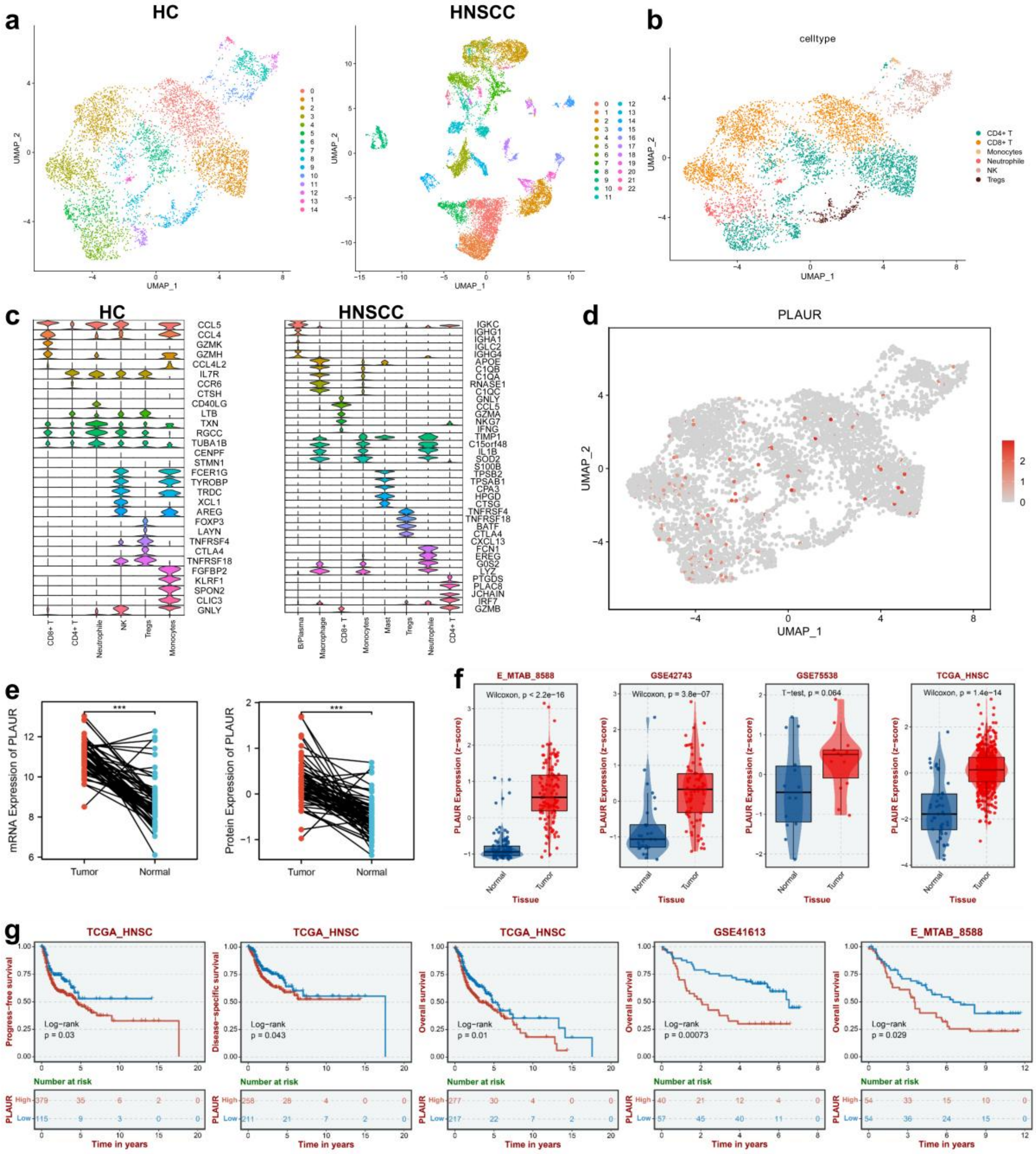

Figure S12. Single-cell transcriptome characterization of immune cells in HNSCC. (a) UMAP dimensionality reduction of immune cells. (b) UMAP of immune cell populations in HC. (c) Marker genes expressed in immune cell populations. (d) Expression of PLAUR is shown in UMAP of immune cell populations in HC. (e) Protein expression and mRNA expression of PLAUR based on CPTAC database. (f) The expression level of PLAUR between HC and HNSCC group. (g) Kaplan-Meier analytical evaluation of the prognostic values of PLAUR. *P < 0.05, **P < 0.01, and ***P <0.001.

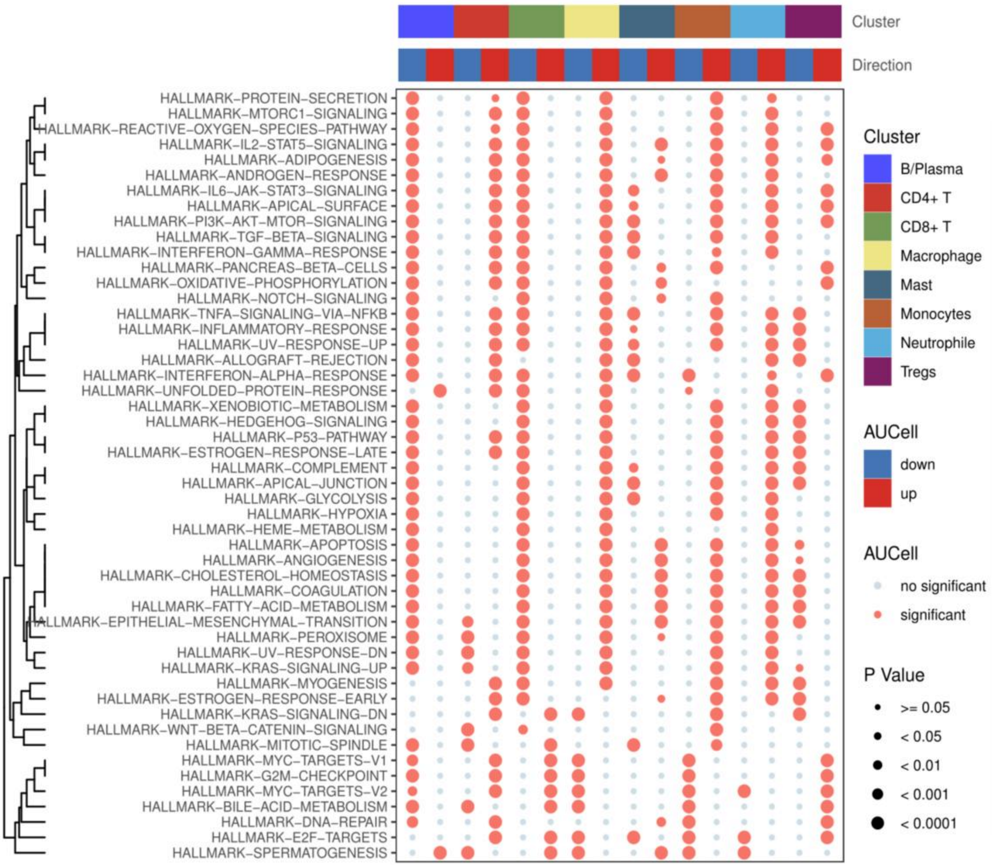

Figure S13. Dot plot showing AUCell scores of signatures among different immune cell clusters.

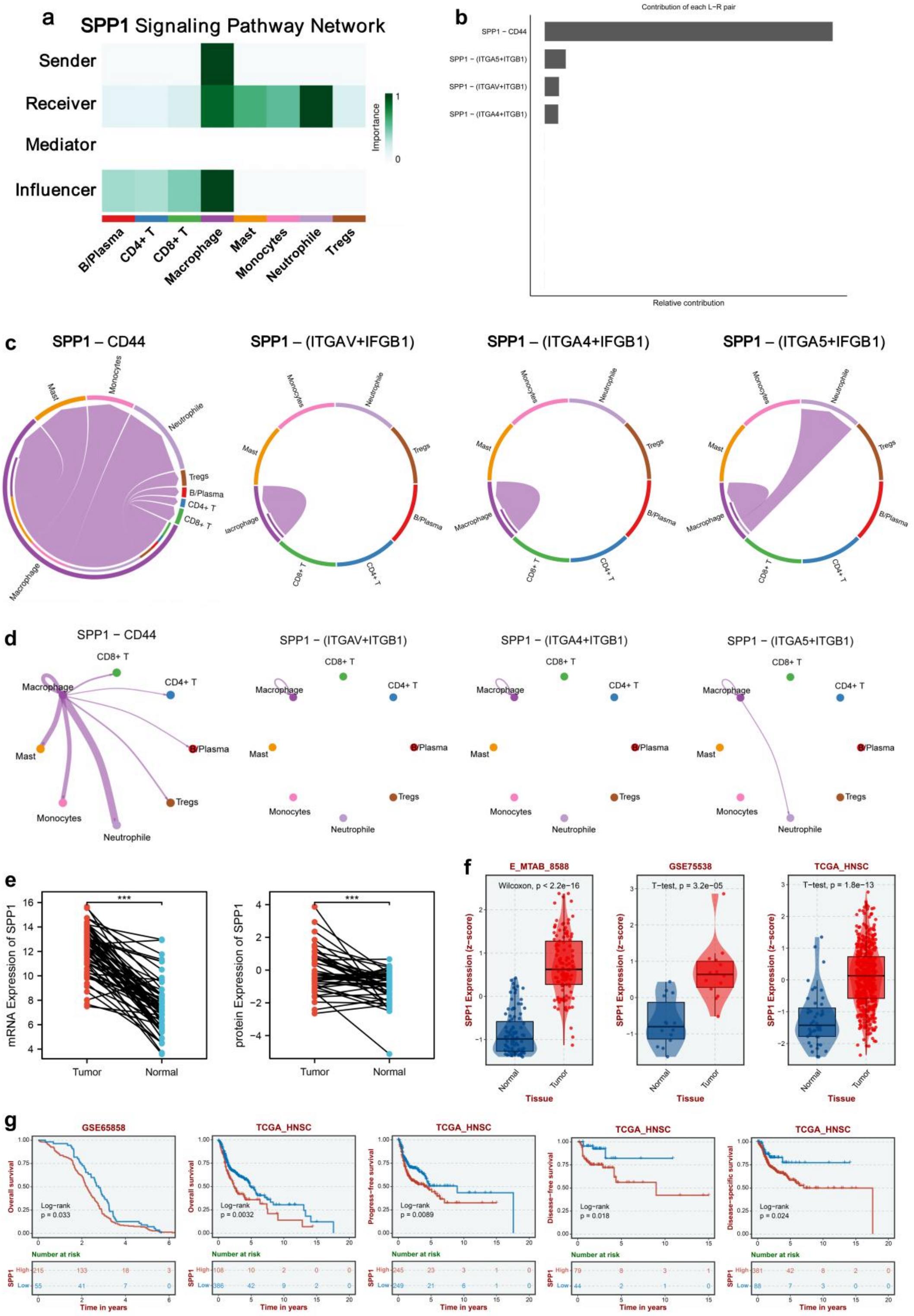

Figure S14. SPP1-related signaling network of immune cells in HNSCC. (a) Heatmap showing communication probabilities from senders (secretors) to receivers (targets) for SPP1 signaling pathway. (b) SPP1-CD44 contributes most to the

interaction among immune cell populations. (c) Chord diagrams of the remaining SPP1-related signaling network of HNSCC immune cell types. (d) Circle plot visualization of the remaining SPP1-related signaling network of HNSCC immune cell types. (e) Protein expression and mRNA expression of SPP1 based on CPTAC database. (f) The expression level of SPP1 between HC and HNSCC group. (g) Kaplan-Meier analytical evaluation of the prognostic values of SPP1. **P* < 0.05, ***P* < 0.01, and ****P* <0.001.

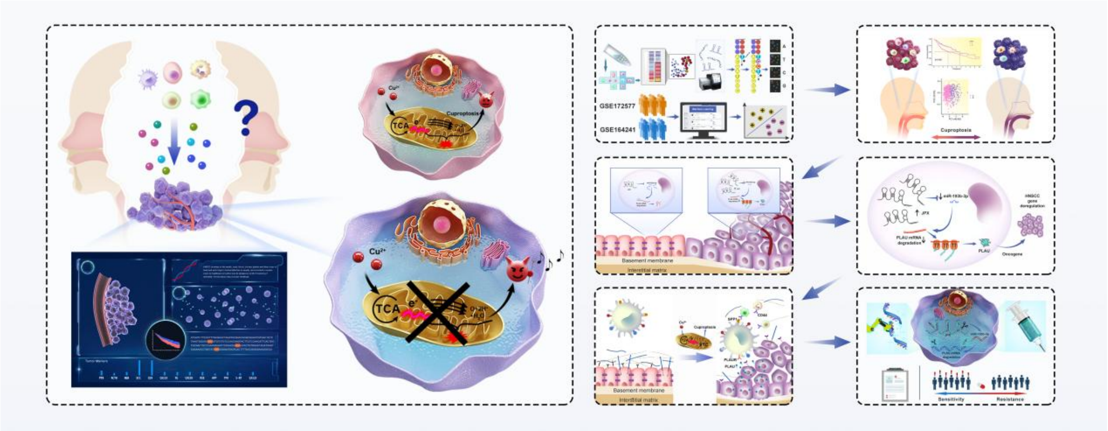

Figure S15. The illustration showed that cuproptosis-related lncRNA JPX regulates malignant cell behavior and epithelial-immune interaction in head and neck squamous cell carcinoma via miR-193b-3p/PLAU axis.

Table S1. The siRNAs and mimics.

	Sequences (5’-3’)
**JPX siRNA**	GGGAACCAGAUGCUGAAAUTT
**miR-193b-3p siRNA**	AGCGGGACUUUGAGGGCCAGUU
**miR-193b-3p mimic**	AACUGGCCCUCAAAGUCCCGCU

Table S2. Primer sequences used for RT-qPCR.

Target genes (rat)	Primer sequences (5'-3')
**JPX**	F:AGACTTAAGATGGCGGCGTT
	R:ACTGGCGAGTTTCTGGACTT
**miR-193b-3p**	F:GTGGTCTCAGAATCGGGGTTT
	R:GACCCCAAAAGCGGGACTT
**PLAU**	F:CGAGCCGCCGTCTAGC
	R:GCAGTTGCACCAGTGAATGT
**STMN1**	F:AGCGGCTTTAGGACCAAACT
	R:CGCGAAGACAGAAGAACCCT
**LAMC1**	F:GCATCTGCATCTCCTACCCC
	R:CGGATGGCTCAGTGTCCTAC
**TGFBR3**	F:TTTCCTCCAACTTGCTGCGG
	R:GGACCTGCAGTGGCTAAACA
**PLAUR**	F:GAGAAGACGTGCAGGGACC
	R:GGCAATCCCCGTTGGTCTTA
**GAPDH**	F:AATGGGCAGCCGTTAGGAAA
	R:GCGCCCAATACGACCAAATC
